# Identification of different mutational profiles in cancers arising in specific colon segments by next generation sequencing

**DOI:** 10.18632/oncotarget.25251

**Published:** 2018-05-08

**Authors:** Duarte Mendes Oliveira, Carmelo Laudanna, Simona Migliozzi, Pietro Zoppoli, Gianluca Santamaria, Katia Grillone, Laura Elia, Chiara Mignogna, Flavia Biamonte, Rosario Sacco, Francesco Corcione, Giuseppe Viglietto, Donatella Malanga, Antonia Rizzuto

**Affiliations:** ^1^ Department of Experimental and Clinical Medicine, University Magna Graecia, Catanzaro, Italy; ^2^ Department of Medical and Surgical Sciences, University Magna Graecia, Catanzaro, Italy; ^3^ Department of Health Sciences, University Magna Graecia, Catanzaro, Italy; ^4^ UOC Chirurgia Generale, Azienda Ospedaliera dei Colli, Napoli, Italy

**Keywords:** colon cancer, ion torrent, colon segments

## Abstract

The objective of this study was to investigate the mutational profiles of cancers arising in different colon segments. To this aim, we have analyzed 37 colon cancer samples by use of the Ion AmpliSeq™ Comprehensive Cancer Panel. Overall, we have found 307 mutated genes, most of which already implicated in the development of colon cancer. Among these, 15 genes were mutated in tumors originating in all six colon segments and were defined “common genes” (i.e. APC, PIK3CA, TP53) whereas 13 genes were preferentially mutated in tumors originating only in specific colon segments and were defined “site-associated genes” (i.e. BLNK, PTPRD).

In addition, the presence of mutations in 10 of the 307 identified mutated genes (NBN, SMUG1, ERBB2, PTPRT, EPHB1, ALK, PTPRD, AURKB, KDR and GPR124) were found to be of clinical relevance. Among clinically relevant genes, NBN and SMUG1 were identified as independent prognostic factors that predicted poor survival in colon cancer patients.

In conclusion, the findings reported here indicate that tumors arising in different colon segments present differences in the type and/or frequency of genetic variants, with two of them being independent prognostic factors that predict poor survival in colon cancer patients.

## INTRODUCTION

Colorectal cancer (CRC) is one of the most commonly diagnosed cancers worldwide [1]. The lifetime risk of CRC in the United States is 6% and the average age at diagnosis is 66 years [2, 3]. In Italy, CRC represents one of the most frequent tumor and the second most frequent cause of cancer mortality, with 19,077 reported deaths in 2011 and 54,000 novel cases expected in 2014 [4].

CRC may have a hereditary component in <5% of cases, being caused by highly penetrant, mendelian cancer syndromes that predisposes to CRC as in familial adenomatous polyposis (FAP) and hereditary non-polyposis colorectal cancer (HNPCC) [5–8]. Conversely, the etiology of the majority of CRCs is sporadic. CRC results from the accumulation of multiple somatic genetic and epigenetic aberrations that lead to the transformation of normal epithelial cells of the intestinal mucosa [9]. According to the model proposed by Fearon and Vogelstein in 1990, CRC proceeds through a series of morphological steps leading from normal mucosa to adenoma and/or carcinoma cells. These morphological changes are caused by specific genetic and/or epigenetic alterations, the first of which is the aberrant activation of the APC/β-catenin pathway, followed by aberrant activation of the RAS/RAF/MAPK pathway due to mutations in genes such as RAS or BRAF and by the loss of p53 function at later stages [10].

Since then, significant progress has been made in the characterization of genetic/epigenetic alterations in CRC [11]. Large-scale sequencing studies have identified numerous recurrently mutated genes (APC, TP53, KRAS, PIK3CA, FBXW7, SMAD4, and NRAS) [12–14] and chromosomal translocations such as the fusions VTI1A–TCF7L2, C2 or f44-ALK, RSPO2 and RSPO3 [15–18]. Within this framework, one of the largest Next generation sequencing (NGS) studies to date, published as part of the Cancer Genome Atlas (TCGA) projects, have identified recurrent alterations within WNT, RAS/MAPK, PI3K, TGF-β and p53 pathways [15]. However, despite the significant progress in defining genetic and epigenetic alterations in CRC [19], we do not yet have a comprehensive understanding of the pathogenesis of different subsets of CRC.

In particular, CRC represents a heterogeneous group of cancer type with rectal cancer presenting marked molecular, morphological and clinical differences with respect to colon cancer [20]. Even tumors arising in different segments of the colon have distinct biological and histological characteristics as well as different outcome, as in the case of right-sided and left-sided colon cancer [21, 22].

In this study, we have applied NGS sequencing to search for different genetic alterations among cancers that originate in six different colon segments. To this aim we have used the Ion AmpliSeq™ Comprehensive Cancer Panel that provides complete exon coverage of the 409 most relevant cancer-associated genes.

By this analysis, we have identified a core of 15 genes that were mutated in tumors originating from all colon segments considered (“common genes”) and 13 genes that were mutated in tumors originating only in one or more specific colon segment (“site-associated genes”). Importantly, mutations in 10 genes (NBN, SMUG1, ERBB2, PTPRD, PTPRT, EPHB1, ALK, AURKB, KDR and GPR124) predicted poor survival, of which none were common genes whereas 1 (PTPRD) was “site-associated genes”.

In particular mutations in ERBB2, PTPRD, PTPRT, AURKB correlated with at least one of the clinical-pathological parameters under analysis such as node status (N), stage, tumor size (T) and/or presence of metastasis (M1).

## RESULTS

### Next generation sequencing of colon cancer samples using the Ion AmpliSeq™ Comprehensive Cancer Panel

The aim of this manuscript was to study the mutational profiles of colon cancers that originated in different colon segments. Patients were selected among those who underwent surgery for colon cancer at the General Surgery Unit of University Magna Graecia of Catanzaro in the years 2013-2015. Samples under analysis derived from different anatomical sites of the colon: ascending colon (n=7), descending colon (n=7), hepatic flexure (n=8), splenic flexure (n=5), transverse colon (n=4) and cecum (n=6). See Supplementary Figure 1 for schematic representation of the different colon segments.

The status of microsatellite instability (MSI) was known for 35 out of 37 tumor samples. Among the 35 samples under analysis two were classified as MSI-High, two were classified as MSI-Low and 31 as MS stable (MSS).

Complete demographics and clinical information, MSI status of patients are reported in Supplementary Table 1 and are summarized in Table 1.

**Table 1:** Demographics of patients (N=37)

Clinical characteristic	N	(%)
**Age (years)**		
Median (Range)	68	(47-84)
**Gender**		
Female	13	(35.1)
Male	24	(64.9)
**Tumor anatomic site**		
Cecum	6	(16.2)
Ascending	7	(18.9)
Hepatic Flexure	8	(21.6)
Transverse	4	(10.8)
Splenic Flexure	5	(13.5)
Descending	7	(18.9)
**TNM grading**		
***T***		
T1	2	(5.4)
T2	5	(13.5)
T3	23	(62.2)
T4	7	(18.9)
***N***		
N0	20	(54.1)
N1	9	(24.3)
N2	7	(18.9)
***M***		
M0	32	(86.5)
M1	4	(10.8)
***Stage***		
I	7	(19.4)
II	13	(36.1)
III	12	(33.3)
IV	4	(11.1)

**Figure 1:**
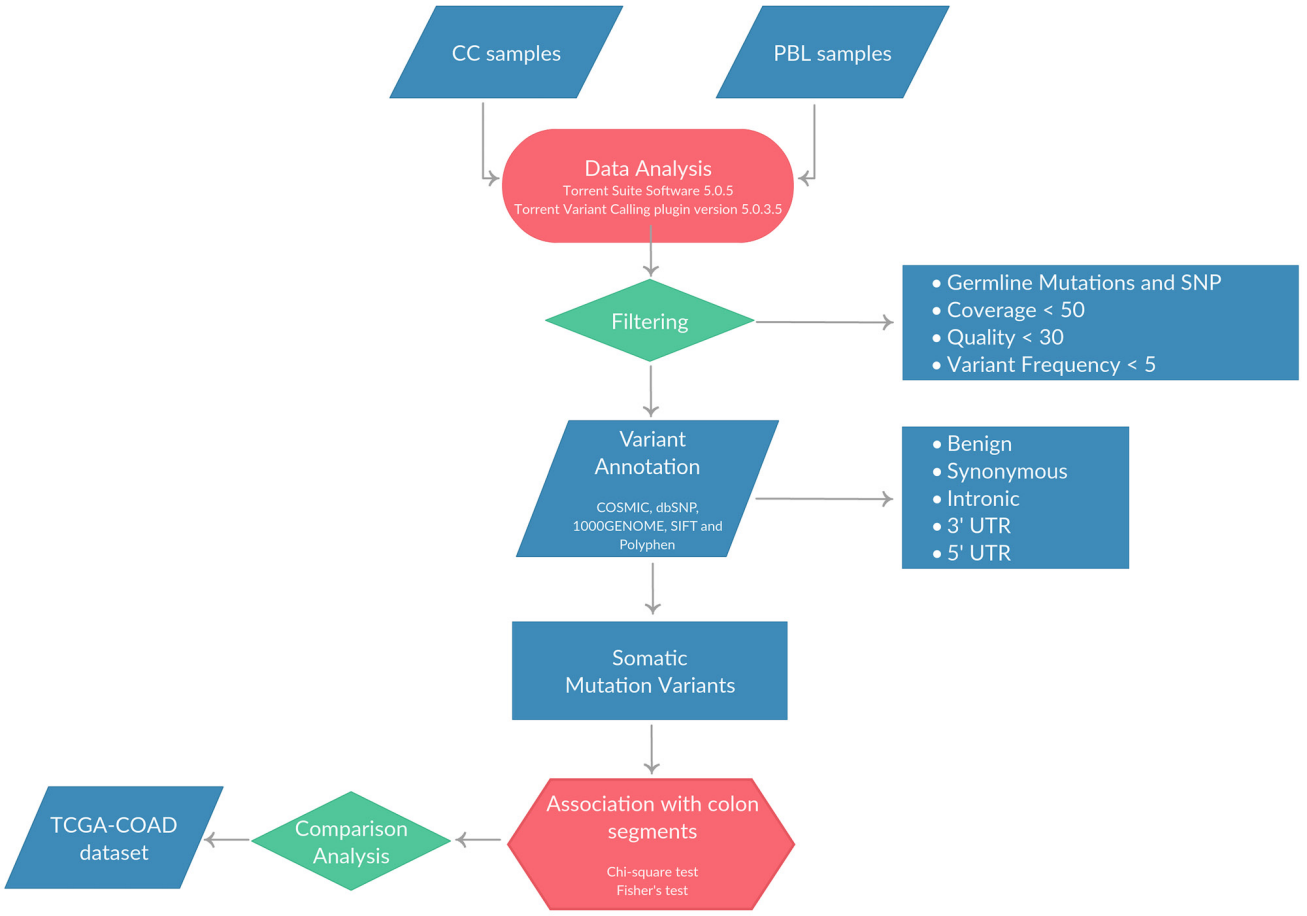
Pipeline applied for the identification of SNVs and/or small indels in the 37 CC patients included in the study

DNA was extracted from matched normal mucosa (n=16) and tumor tissues (n=37) and quantified as described in Materials and Methods. Where available, DNA was extracted also from peripheral blood of the same patients (PBL, n=13). Once checked for quality, NGS was performed using the Ion Torrent platform with the Ion AmpliSeq™ Comprehensive Cancer Panel (CCP). The mean number of reads/sample generated was 13×10^6^. The mean sequence coverage depth was 777 (range 102.5-2,656), with median uniformity of sequenced genes being of 97% (range 70–99%). The average number of variants identified in tumor samples after quality and coverage filtering was 1,658 (range 593-14,448), the average number of variants identified in normal mucosa was 1,108 (range 575-1,279) and the average number of variants identified in PBL was 1,109 (range 904-1,268). Single-nucleotide variants (SNVs) and small insertions and/or deletions (indels) were predicted using the algorithms described in Materials and Methods. All synonymous variants as well as those present within 5’UTR and 3’UTR were not considered in this study. The analysis workflow is reported in Figure 1.

In order to select the true somatic variants, each tumor was compared with the corresponding paired normal mucosa or PBL, when available. Surprisingly, we found that some of the normal mucosa presented multiple variants that were detected also in the corresponding tumor but not in the PBL (i.e. APC, KRAS, TP53) (data not shown). This result is in agreement with a recent manuscript showing the occurrence of CNAs as a common feature of the histologically normal tissue from CRC patients [23]. For this reason, the filtering of germline variants, for the identification of true somatic mutations, was performed by pooling the variants detected in the 13 PBL samples, generating a “virtual pool” of germline variants as previously described [24–27]. Somatic variants were further filtered using public databases (dbSNP141 and the 1000 Genomes). Finally, we selected non-synonymous variants within the coding regions of the genes that were predicted to be damaging by both SIFT and Polyphen2 algorithms.

**Figure 2:**
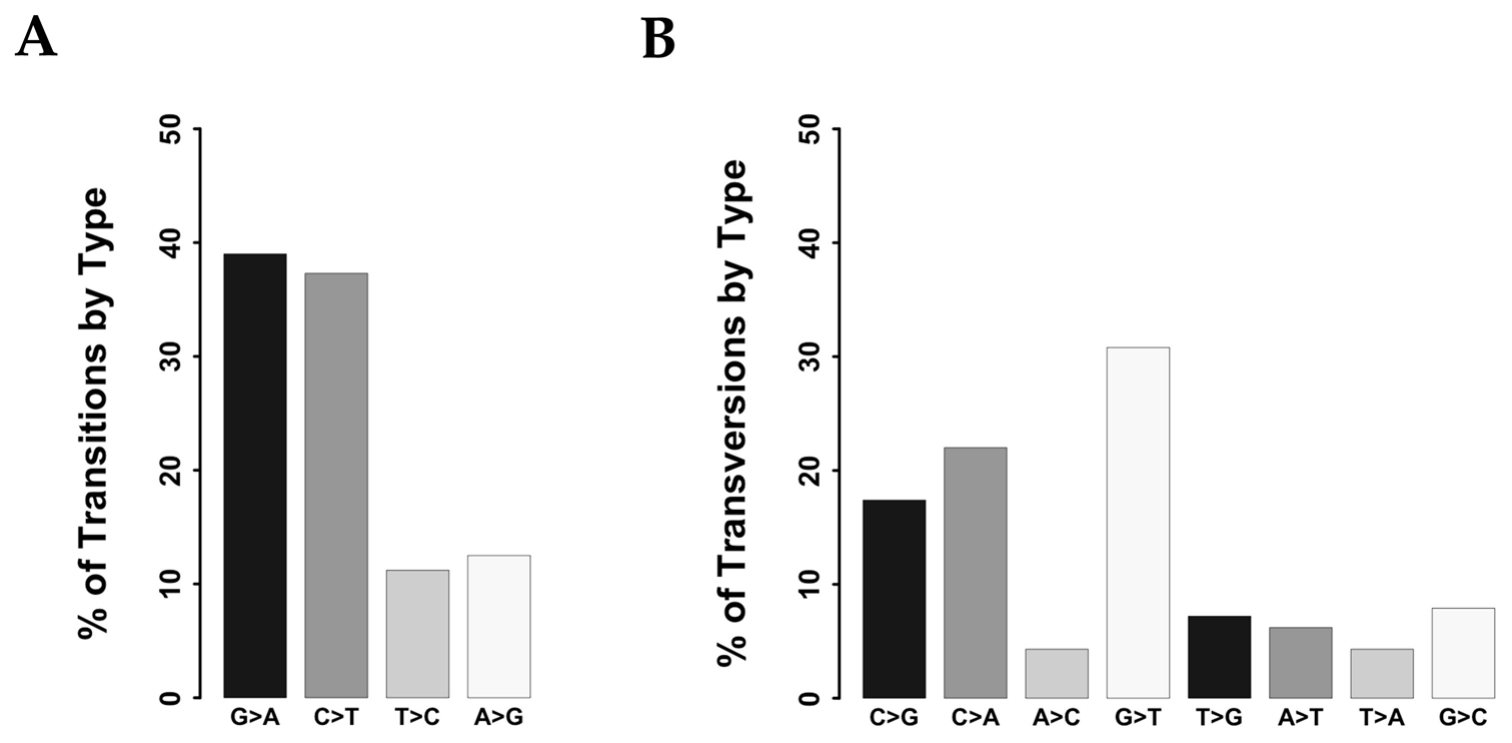
Classification of variants identified in CC samples **(A)** Bar chart showing the distribution of transitions. **(B)** Bar chart showing the distribution of transversions.

Overall, we identified 1,385 potential protein-altering somatic variants (see Supplementary Table 2) distributed within 307 genes among the 37 colon cancer samples. The number of variants/tumor ranged from 4 to 112, with a median value of 30. Total COSMIC variants were 58 (see Supplementary Table 3), with a median value of 1.57 COSMIC variant/tumor (range 0-7). Each tumor presented a median number of 24 mutated genes (range 3-96). Identified SNVs included 703 transitions and 305 transversions. Transitions were distributed as follows: 262 C>T (37.3%), 79 T>C (11.2%), 88 A>G (12.5%) and 274 G>A (39%) (Figure 2A) whereas transversions were: 67 C>A (22%), 13 A>C (4.3%), 94 G>T (30.8%), 22 T>G (7.2%), 19 A>T (6.2%), 13 T>A (4.3%), 24 G>C (7.9%) and 53 C>G (17.4%) (Figure 2B). The distribution of transitions and transversions in tumors stratified by colon segments are reported in Supplementary Figures 2-7.

Results obtained by NGS were confirmed through Sanger DNA sequencing performed on matched PBL and cancer samples (CC12 and CC28). Representative sequencing analysis of BRAF and APC variants is shown in Supplementary Figure 8. In conclusion, the analysis of 37 CC samples by CCP allowed the identification of 1,385 non-synonymous variants in 307 genes. As a further control we compared the results obtained with the CCP to the sequencing data present in public databases (COAD-TCGA). The analysis of COAD-TCGA dataset was restricted to the 409 cancer-related genes and led to the identification of mutations in 397 genes. Conversely, the median value of damaging variants/tumor present in COAD-TCGA dataset was 14 (range 1–206) whereas it was 30 (range, 4-112) in the 37 CC. This discrepancy may be ascribed to the higher depth of analysis reached by using the CCP in comparison with exome or whole genome sequencing reported for samples present in the COAD-TCGA.

A further control was to investigate whether the cohort of samples analyzed in this study reflected the well-known distinction between hypermutated and non-hypermutated colorectal tumors. Accordingly, hypermutated tumors were defined as those showing a mutation rate higher than 12 mutations/10^6^ base pairs whereas non-hypermutated tumors were those with mutation a rate <8.24 mutations/10^6^ base pairs [15]. Therefore, we re-analysed the data relative to the 37 CC samples on the basis of these thresholds. Mutation analysis of the 409 cancer-related genes present in the CCP in the 37 CC samples under study revealed that 81% of the samples was hypermutated, at difference with the value of 15% deriving from an analysis of the pre-existing literature [15]. This is likely due to a bias in the number and type of genes present in the CCP. In fact, mutation rate thresholds have been established using whole exome sequencing whereas our analysis as performed on 409 genes selected to be implicated in cancer. Accordingly, limiting the analysis of mutation rate in tumors present in TGCA to the 409 genes of CCP revealed a high frequency of hypermutated tumors (63%).

### Mutations in genes commonly mutated in colon cancer

**Figure 3:**
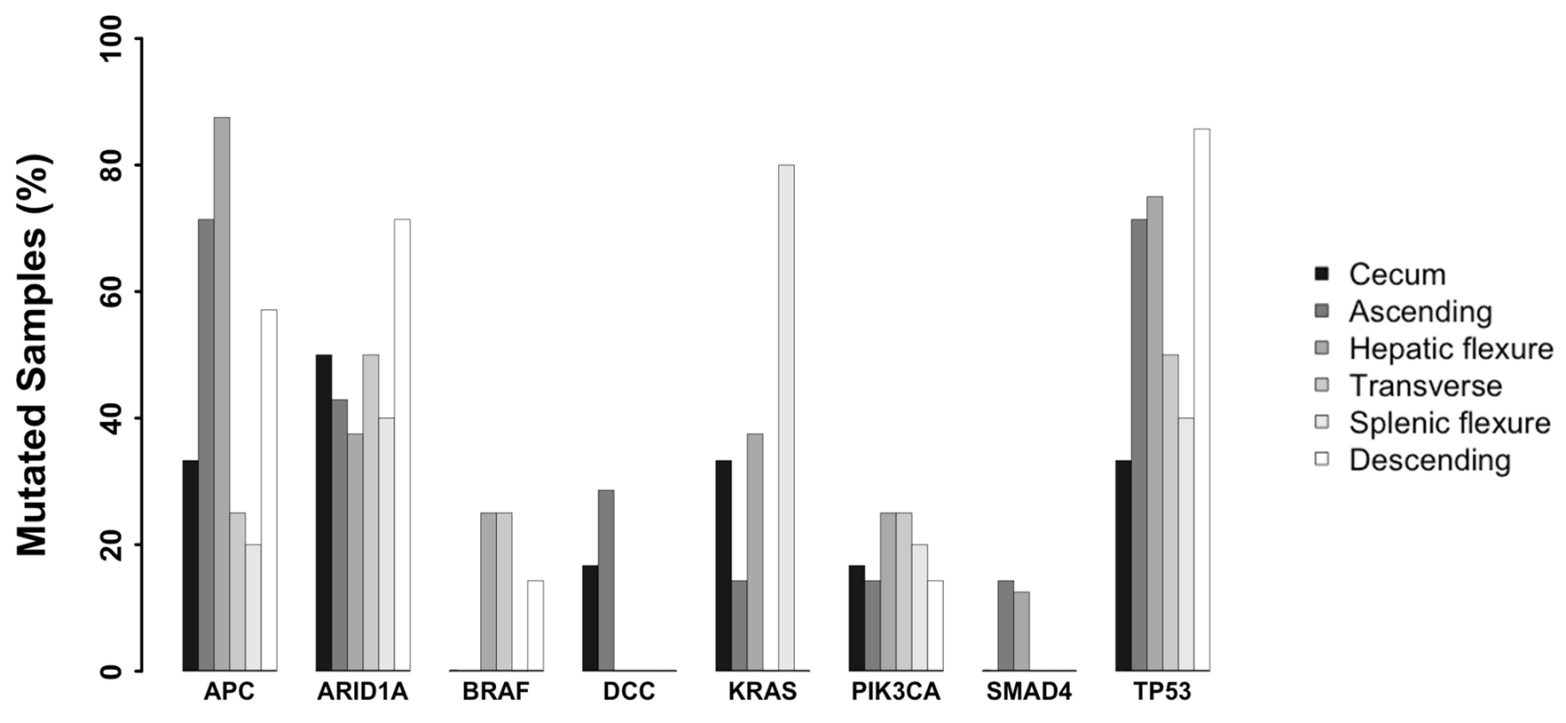
Frequency of variants identified in genes recurrently associated with CC stratified by colon segments Bar plot showing the frequency of variants in eight genes recurrently associated with CC stratified by colon segments.

First we investigated the presence and the frequency of mutations in 8 genes commonly mutated in colon cancer in the cohort of patients under analysis: APC was mutated in 54% of samples, TP53 in 62% of samples, PI3KCA in 20% of samples, ARID1A in 48.6% of samples, KRAS in 27% of samples, BRAF in 11% of samples, DCC in 8% of samples and SMAD4 in 5.4% of samples. See Figure 3 for the distribution of commonly mutated genes in tumors originating in different colon segments and Supplementary Figure 9.

We identified 20 different variants in APC distributed in 20 samples, of which 10 were present in the COSMIC database (Supplementary Table 3). Expectedly, the majority of the identified SNVs were loss-of-function variants localized in critical functional domains of the APC gene. See Mutation mapper plots [28] in Supplementary Figures 10-11.

We also evaluated the correlation between APC mutations and MSI status. APC mutations were found in 3/4 MSI tumors but we failed to find significant association between mutations in APC and the MSI status.

As to TP53, 18 different variants were identified across 23 colon cancer patients, of which 11 were present in the COSMIC database (see Supplementary Table 3). All identified variants fell within the p53 DNA binding domain, except P47fs (see Mutation mapper plots in Supplementary Figures 10-11).

Variants in the ARID1A gene, which encodes a key component of the highly conserved SWI–SNF (switch/sucrose non-fermentable) chromatin remodeling complex, were identified in 18/37 colon cancer patients with 13 different variants. See Mutation mapper plots in Supplementary Figures 10-11.

As to the gene encoding the catalytic subunit of PI3K (PIK3CA), 5 different gain-of-function variants were identified in 7/37 samples (20%), of which 3 were present in the COSMIC database (Supplementary Table 3). Most variants with confirmed oncogenic potential occurred in the helical domain encoded by exon 9 (E542K, E545A, E545G and E545K). Importantly, these variants have been recurrently reported in colon cancer [29, 30] (see mutation mapper plots in Supplementary Figures 10-11. Five different variants (G12V, G12D, G12S, G13D, A146V) were identified in KRAS (10/37 patients), all of which were present in the COSMIC database. Conversely, only two COSMIC variants were identified in BRAF (D594G and V600E) in 4/37 patients and 4 novel nonsense variants were identified in DCC (3/37 patients). In line with existing literature, we found that BRAF mutations were mutually exclusive with KRAS mutations.

Finally, 2 variants were identified in SMAD4: the missense R361H variant, present in the COSMIC database, and the novel nonsense Q239^*^ variant (Supplementary Table 3 and Supplementary Figures 10-11.

### Clinical-pathological correlations

We correlated the presence of SNVs in a specific gene and/or group of genes with at least 5% of mutated samples (3 on 35) with clinical and pathological parameters of the patients under analysis. We found that multiple genes, when mutated, correlated with overall survival (OS), node status, stage, tumor size and presence of metastasis (*p*-value < 0.05). See Supplementary Table 4.

**Figure 4:**
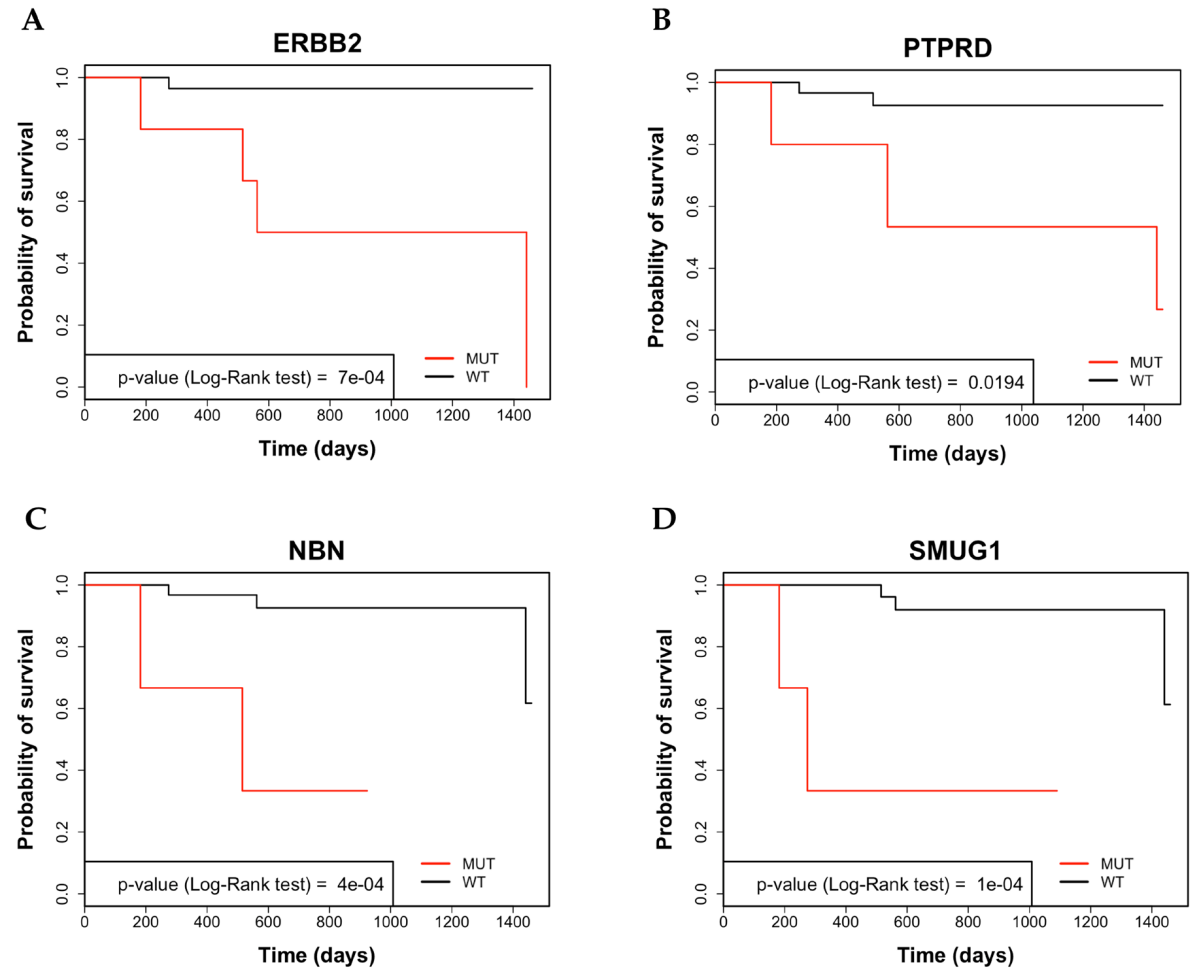
Analysis by Kaplan-Meier curve of 4-year survival in CC patients **(A)** Kaplan-Meier survival curve of overall survival (OS) in CC patients that were stratified for the presence of variants in ERBB2. **(B)** Kaplan-Meier survival curve of OS in CC patients that were stratified for the presence of variants in PTPRD. **(C)** Kaplan-Meier survival curve of OS in CC patients that were stratified for the presence of variants in NBN. **(D)** Kaplan-Meier survival curve of OS in CC patients that were stratified for the presence of variants in SMUG1.

The average 4-year survival rate of all colon cancer patients under analysis was 86%. Mutations in NBN, SMUG1, ERBB2, PTPRD, PTPRT, EPHB1, ALK, AURKB, KDR and GPR124 genes were associated with poor OS. See representatives Kaplan Meier curves in Figure 4 and Supplementary Figure 12. The 4-year survival rate of patients negative for mutations in these genes was 91.3% (range 89.6% - 96.5%), conversely, the 4-year survival rate of patients showing mutations in the same genes ranged from 33.3% for NBN, SMUG1, ERBB2, EPHB1 and AURKB to 66.6% in the case of GPR124).

Subsequent univariate Cox Regression Analysis revealed significant T, M and stage as well as mutations in NBN, SMUG1, ERBB2, PTPRT, EPHB1, ALK, PTPRD, AURKB, KDR and GPR124 were predictors of poor OS. See Table 2.

**Table 2:** Univariate Cox regression analysis between genes showing SNVs and patients’ OS or clinical covariates previously selected by Log-Rank test in CC patients

Covariates	OS		
	HR	95% CI	*p*-value
**T (TMN) (1-3/4)**	12.33	1.28 - 118.8	0.029^*^
**Stage (I+II+III/IV)**	0.1175	0.01 - 0.73	0.0221^*^
**M (TNM) stage (M0/M1)**	11.7674	1.83 - 75.52	0.00935^*^
**NBN**	14.75	2.03 - 106.8	0.0004^*^
**SMUG1**	17.754	2.42 - 129.8	0.0001^*^
**ERBB2**	17.158	1.8 - 156.2	0.0007^*^
**PTPRT**	11.342	1.57 - 81.77	0.002^*^
**EPHB1**	11.005	1.54 - 78.25	0.002^*^
**ALK**	7.49	1.05 - 53.21	0.01^*^
**PTPRD**	8.0739	1.18 - 54.9	0.019^*^
**AURKB**	8.689	1.16 - 64.62	0.02^*^
**KDR**	6.897	0.96 - 49.11	0.025^*^
**GPR124**	6.853	0.94 - 49.78	0.028^*^

^*^p-value ≤ 0.05.

To determine whether the factors identified in univariate analysis were independent predictors of OS we performed multivariate analysis. Mutations in NBN and SMUG1 resulted to be independent predictors of poor OS. The OS of patients harbouring mutations in NBN or SMUG1 was 33.3%, compared to the value of 90.6% shown by patients without mutations in these genes. See Table 3.

**Table 3:** Multivariate Cox regression analysis

Multivariate analysis			
Covariates	OS		
Model 1	HR	95% CI	p-value
**SMUG1**	306.07	11.261668 - 95261.724	0.0003
**T (TMN) (1-3/4)**	16	1.514606 - 2216.416	0.01
**Model’s likelihood ratio test p-value = 0.0001**			

Model 2	HR	95% CI	p-value
**NBN**	18.47	2.252757 - 212.3302	0.008
**M (TNM) stage (M0/M1)**	13.38	1.967101 - 144.2825	0.008
**Stage (I+II+III/IV)**	13.38	1.967101 - 144.2825	0.008
**Model’s likelihood ratio test p-value = 0.00123**			

Notably, NBN mutated patients showed a mean survival time of 21months (SE 8.08 months) in comparison with NBN-negative patients (38 months, SE 1.3 months), whereas SMUG1 mutated patients showed a mean survival time of 19 months (SE 9.5 months) in comparison with SMUG1-negative patients (41 months, SE 1.3 months). It is necessary to underline that although mutated ERBB2, PTPRT, PTPRD and AURKB predicted poor OS in univariate analysis they were excluded from the multivariate analysis because they were preferentially identified in patients with late stage (stage III-IV) and/or metastatic disease.

### Colon cancers arising in different anatomical segments present different combinations of mutated genes

Colorectal cancer is an heterogeneous disease with tumors arising in the right segment of the colon presenting molecular, morphological and clinical differences with respect to tumors arising in the left segment of the colon or in the rectum [22, 30].

In this manuscript, we performed a more extensive analysis in order to define mutation patterns in tumors arising in different colon segments (ascending colon, descending colon, transverse colon, hepatic flexure, splenic flexure and cecum). This allowed the identification of a site-associated pattern of genetic alterations based on tumor site.

We demonstrated that tumors originating in certain segments presented higher median number of mutated genes compared to tumors originating in other segments. For example, tumors originating in the ascending colon presented a number of mutated genes (mean=37) significantly higher than tumors originating in cecum (mean=15, p.value=0.05). Concerning the number of mutations that tumors originating in the ascending colon (mean= 44) presented a number of variants significantly higher than tumors originating in cecum (mean= 18), in particular the number of missense variants (mean= 30) was also significantly higher (mean= 11) and the number of frameshift mutations was higher in ascending colon and hepatic flexure tumors compared with splenic flexure tumors (10, 10 and 4; p-value=0.03 and 0.01 respectively). See Supplementary Figure 13.

In order to define specific mutation patterns in tumors arising in different colon segments we stratified the mutated genes identified by the CCP analysis reported in this manuscript according to tumor localization within the colon. To this aim we define two different sub-groups of mutated genes, “common genes” and “site-associated” genes.

Genes that presented mutations in at least one tumor deriving from all 6 colon segments were defined “common genes” whereas “site-associated” genes were defined as those that were preferentially mutated in one or more specific colon segments.

**Figure 5:**
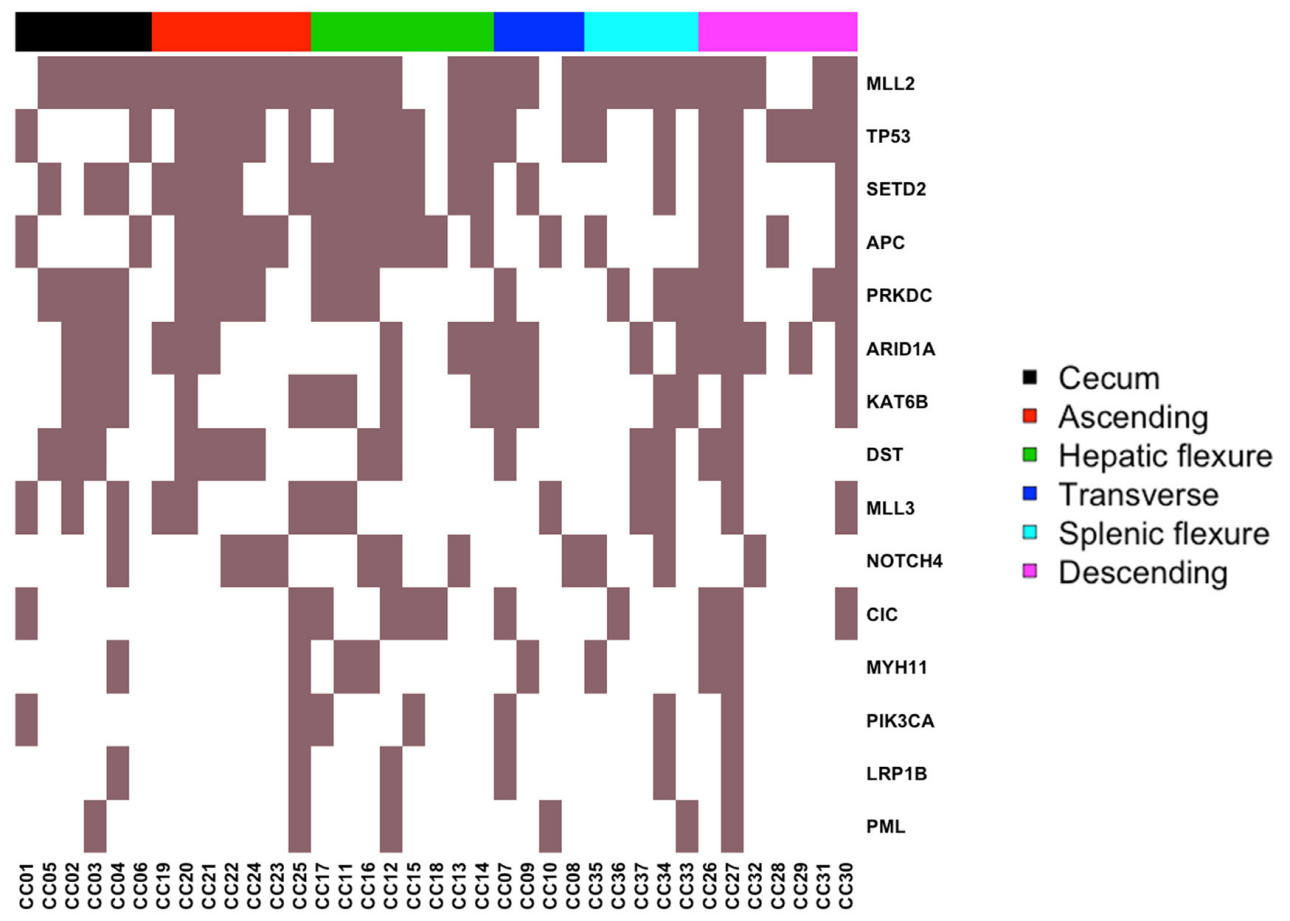
Heat-map representing the distribution of common mutated genes Columns represent the 37 CC included in the study grouped by colon segment; rows represent mutated genes.

Among mutated “common genes” were APC, ARID1A, CIC, DST, KAT6B, LRP1B, MLL2, MLL3, MYH11, NOTCH4, PIK3CA, PML, PRKDC, SETD2 and TP53. The list of mutated “common genes” and the corresponding mutation frequencies are reported in the heat-map of Figure 5 and in Table 4. Position and amino acid changes of are reported in the Mutation Mapper plots. See Supplementary Figures 14-16. Interestingly, several mutated “common genes” including APC, TP53, PIK3CA, ARID1A and MLL3 have already been implicated in the development of colon cancer. On the other hand, CIC, DST, KAT6B and NOTCH4 represent novel genes that may be implicated in the onset and/or development of colon cancer.

**Table 4:** "Common mutated" genes stratified by colon segments (%)

Symbols	Cecum	Ascending colon	Hepatic flexure	Transverse colon	Splenic flexure	Descending colon
**TP53**	33	71	75	50	40	86
**ARID1A**	50	43	38	50	40	71
**MLL2**	83	100	75	75	100	71
**APC**	33	71	88	25	20	57
**PRKDC**	67	57	38	25	60	57
**CIC**	17	14	50	25	20	43
**SETD2**	50	71	88	25	20	43
**DST**	50	57	25	25	40	29
**KAT6B**	50	29	50	50	40	29
**MLL3**	50	43	25	25	40	29
**MYH11**	17	14	25	25	20	29
**LRP1B**	17	14	12	25	20	14
**NOTCH**	17	43	38	25	40	14
**PIK3CA**	17	14	25	25	20	14
**PML**	17	14	12	25	20	14

Notably, we found a high number of inactivating mutations in a sub-group of “common gene” that encode chromatin remodelling regulators such as MLL3, MLL2, KAT6B and SETD2. MLL3 presented 11 different mutations (10 missense and one frameshift), MLL2 presented15 different mutations (11 missense, 3 frameshift deletions and 1 nonsense), KAT6B presented 4 different mutations (1 missense, 2 frameshift deletions, 1 in frame insertion) and SETD2presented 12 different mutations (9 missense, 2 frameshift deletions and 1 nonsense).

On the other hand, we found that 13 genes, when mutated, resulted associated to a specific colon segment, and were defined “site-associated” genes. Given the small size of the sample under study, we performed a two-step statistical analysis that included a prior analysis of the associations by the Chi-square test followed by individual validation with Fisher’s test that is more appropriate for small size samples (significance set at p-value<0.05). See Table 5 and Figure 5.

**Table 5:** “Site-associated” mutated genes listed by p-value

GENE Symbol	p-value	Colon segments					
		Ascending (N=7)	Cecum (N=6)	Descending (N=7)	Hepatic flexure (N=8)	Splenic flexure (N=5)	Transverse (N=4)
**CBL**	0.001	4 (57.14%)	0	0	0	0	0
**LAMP1**	0.003	0	0	0	0	0	2 (50%)
**PHOX2B**	0.01	0	0	0	0	2 (40%)	0
**ATM**	0.01	0	0	0	0	2 (40%)	0
**FGFR2**	0.01	0	0	0	0	2 (40%)	0
**GRM8**	0.01	0	0	0	0	2 (40%)	0
**FLT4**	0.02	5 (75.43%)	0	2 (28.57%)	2 (25%)	0	0
**PTPRD**	0.02	1 (14.29%)	0	0	0	3 (60%)	1 (25%)
**PPARG**	0.02	1 (14.29%)				3 (60%)	1 (25%)
**BLNK**	0.02			3 (42.86%)	3 (37.50%)	4 (80%)	1 (25%)
**KRAS**	0.03	1 (14.29%)	2 (33.33%)	0	3 (37.5%)	4 (80%)	0
**COL1A1**	0.03	0	0	4 (57.14%)	1 (12.5%)	1 (20%)	
**NOTCH1**	0.04	4 (57.14%)	0	2 (28.57%)	0	1 (20%)	0

Mutations in PHOX2B, ATM, PTPRD, BLNK, FGFR2, GRM8, KRAS and PPARG were preferentially detected in tumors arising in the splenic flexure; mutations in LAMP1 was preferentially identified in tumors arising in the transverse colon; mutations in CBL, NOTCH1 and FLT4 were found preferentially in tumors arising in the ascending colon and mutations in COL1A1 were found preferentially in tumors arising in the descending colon. However, the majority of the identified variants were not present in the COSMIC database and thus must be considered of uncertain significance. The position of the identified mutations is reported in Supplementary Figures 17-19.

Finally, from the analysis presented in Supplementary Table 4 that reports the association between the presence of mutations and clinical pathologic parameters a significant correlation was found between the “site-associated” genes PHOX2B, ATM, PTPRD and COL1A and stage, T and and/or M. In addition, as indicated above, PTPRD was shown to predict poor OS.

## DISCUSSION

In this study we have investigated the mutational profile of cancer that originated in six different segments of the colon that include ascending colon, descending colon, hepatic flexure, splenic flexure, transverse colon and cecum. The main results comprise: i) the identification of 1,385 potential protein-altering somatic variants in 307 different genes, with a median number of 24 mutated genes/tumor (range 3-96), ii) the identification of 15 genes that were mutated at least once in tumors originating in all colon segments; iii) the identification of 13 genes that were preferentially mutated in tumors originating in specific colon segments, iv) the identification of 10 genes (NBN, SMUG1, ERBB2, PTPRD, PTPRT, EPHB1, ALK, AURKB, KDR and GPR124) that were associated with node status, stage, tumor size and/or presence of metastasis and that predicted poor survival, v) the finding that mutations in two genes (NBN and SMUG1) were independent predictors of survival.

In the multistep genetic model of colorectal carcinogenesis proposed by Fearon and Vogelstein, multiple genetic events are apparently responsible for onset and progression of CRC [31]. Although the original model predicted that at least 7 distinct mutations were required for cancer development, recent NGS studies have identified mutations in > 80 genes in CRC, though less than 15 genes were effectively considered to drive colon tumorigenesis [14, 32].

The analysis reported here allowed the identification of 307 cancer-associated genes with potential protein-altering somatic variants. In agreement with previous studies, the median number of mutated genes/tumor observed in the cohort of patients described here was 24. However, the number of driver genes that effectively drive colon tumorigenesis remains to be determined. Colon cancer represents a heterogeneous group of neoplasms that presents marked molecular, morphological and clinical differences. Accordingly, tumors arising in different segments of the colon (i.e. right-sided, left-sided) have distinct biological and histological characteristics as well as different outcome [21, 22]. In this study, we have expanded the classical division of tumors originating in the right or in the left colon and applied NGS sequencing to search for genetic alterations in cancers that originate in six segments (ascending colon, descending colon, hepatic flexure, splenic flexure, transverse colon and cecum).

On the basis of the data generated in this manuscript we selected two groups of mutated genes for further analysis. The first group comprises 15 genes that were mutated at least once in tumors originating in all colon segments under analysis and were defined “common genes”. The second group comprises 13 genes that were mutated preferentially in tumors originating in one or more colon segment and were defined “site-associated genes”. Mutated “common genes” comprise APC, ARID1A, CIC, DST, KAT6B, LRP1B, MLL2, MLL3, MYH11, NOTCH4, PIK3CA, PML, PRKDC, SETD2 and TP53 whereas mutated “site-associated genes” comprise PHOX2B, ATM, PTPRD, BLNK, FGFR2, GRM8, KRAS and PPARG, LAMP1, CBL, COL1A1, NOTCH1 and FLT4.

Malignant transformation of the colon epithelium is driven by early inactivation of tumor suppressor APC, followed by mutations of KRAS/BRAF, PIK3CA and TP53 [10, 33–36]. In agreement with this notion, we have found frequent mutations in APC, KRAS, PIK3CA, BRAF and TP53. Mutation frequency of these genes was similar to what reported in the literature for TP53 (60%) and lower for APC, KRAS, PIK3CA and BRAF, likely because of the limited number of samples analyzed in this study. Expectedly, variants in APC, TP53, ARID1A, SMAD4 and DCC were predicted to be inactivating mutations whereas variants identified in KRAS, BRAF and PIK3CA were apparently gain-of-function. In agreement with the literature, KRAS mutations were mutually exclusive with BRAF mutations [37].

Tumors arising in different colon segments presented multiple previously unrecognized differences in the type and/or frequency of genetic variants. As anticipated, we identified 15 “common genes” and 13 “site-associated genes” The remaining 279 mutated genes were not significantly associated with a specific colon segment tumor. The finding that cancers that originated in specific colon segments presented different mutational profiles suggests that they are apparently caused by distinct molecular alterations. Accordingly, mutations in APC, TP53 and PI3KCA were present in tumors originating in all colon segments whereas mutated KRAS was apparently among “site-associated genes”.

Expectedly, most relevant “common genes” have already been implicated in the development of colon cancer. In fact, loss-of-function mutations in APC, TP53, ARID1A, MLL2 and MLL3 as well as gain-of-function mutations of PIK3CA represent well-known genetic alterations that contribute to the process of colon carcinogenesis [38–40]. Among the mutated “common genes”, a special comment on the epigenetic regulators MLL2 and MLL3 is warranted. Epigenetic alterations play a pivotal role in the inactivation of tumor suppressor genes and activation of oncogenes in a wide spectrum of tumors including CRC. Alteration of the chromatin status is known to be an early event during the malignant transformation of colonic epithelial cells [41]. Notably, MLL2 and MLL3 are methyl-transferases that methylate the Lys-4 position of histone H3 and thus represent key transcriptional regulators that are essential for cell differentiation, embryonic development, cell fate transition [42] metabolism [43] and tumor suppression [44].

Our results indicated that mutations in MLL2 and MLL3 are frequently detected in colon cancer samples. These data are in agreement with the finding that MLL2 is one of most frequently mutated gene in lymphoma, medulloblastoma as well as in prostate, bladder, gastric and lung carcinomas [45–48]. Most detected alterations in MLL2 and MLL3 include frameshift and/or nonsense changes, suggesting that they are loss-of-function mutations and thus a role of tumor suppressors for MLL2 and MLL3. Accordingly, the mutations detected in MLL2 and MLL3 identified in cancer thus far are apparently loss-of-function mutations [49, 50]. On the other hand, CIC, DST, KAT6B and NOTCH4 have not been previously implicated in the development of colon cancer, and for this reason, deserve further studies.

Our results indicate also that cancers that originate in specific colon segments are apparently caused by distinct molecular alterations. The most frequently mutated “site-associated gene” is BLNK. BLNK encodes a cytoplasmic linker protein downstream of the B-cell receptor that plays a critical role in B cell development [51]. Its deficiency is associated with a high incidence of spontaneous pre–B-cell lymphoma, acting as a tumor suppressor [52]. In addition, BLNK mutations have been frequently identified also in multiple solid cancers [53, 54]. The results presented in this study indicated that BLNK mutations occurred preferentially in tumors originating in the splenic flexure. In agreement with published data most of BLNK mutations were frameshift and/or nonsense, thus causing premature truncation of the protein.

Another “site-associated gene” identified in this study is PTPRD, a gene that encodes a tyrosine-protein phosphatase that is preferentially mutated in tumors originating in the splenic flexure of the colon. In addition, the presence of PTPRD mutations predicted poor prognosis. Homozygous deletions and/or epigenetic silencing of PTPRD have also been identified in multiple human cancers, including glioblastoma, lung carcinoma and head and neck carcinoma [55], indicating that PTPRD is a tumor-suppressor gene [56, 57]. PTPRD regulates adhesion and migration of cancer cells in cooperation with β-catenin/TCF signaling, and its loss promotes cancer progression [58].

Notably, mutations in 9 additional genes such as NBN, SMUG1, ERBB2, PTPRT, EPHB1, ALK, AURKB, KDR and GPR124 predicted poor survival. Among these, ERBB2, PTPRD, PTPRT, AURKB also correlated with node status, stage, tumor size and/or presence of metastasis.

Although the genes predicting poor survival that were identified in this study were 10, only mutations in NBN and SMUG1 turned out to be significant in multivariate analysis and thus can be considered independent prognostic factors for colon cancer patients. Notably, both genes are implicated in the molecular mechanisms that regulate DNA repair. NBN is a central player in non-homologous and homologous recombination repair [59] whereas SMUG1 encodes a uracil DNA glycosylase implicated in base excision repair [60] and is apparently associated with resistance to 5-FU based and/or radio-therapy [61]. Somatic mutations in NBN and SMUG1 in cancer (CC) have not been reported so far, though NBN germ-line mutations predispose to increased risk of developing cancer [62]. Identified NBN mutations include nonsense Q279^*^ that cause a premature truncation of the protein and missense V210F and V242A that fall in the BRCT domain necessary for recognition and repair of double strand breaks [62]. Conversely, identified SMUG1 mutations (Q93K, R187^*^ and R220W) fall in the catalytic domain. These novel findings suggest that inactivating mutations in these genes may contribute to the genomic instability that characterizes a subset of colon cancer samples.

In conclusion, the findings reported above indicate that tumors arising in different colon segments presented interesting unrecognized differences in the type and/or frequency of genetic variants. Colon cancer develops as the consequence of the accumulation of mutations in a small group of “common genes” that include APC, PIK3CA, TP53, and, depending on the different localization in which they arise, of mutations in “site-associated” genes such as BLNK and PTPRD. Notably, 10 genes, when mutated, were found to be clinically relevant, predicting poor survival. In particular, NBN and SMUG1 turned out to be independent prognostic factors that may predict response to therapy. However the conclusions drawn from this study need further validation due to the relatively small number of samples analyzed.

## MATERIALS AND METHODS

### Ethics statement

Patient accrual was conducted according to Institutional Review Board of the AOU Mater Domini/University Magna Graecia (Catanzaro, Italy). The study was approved by the Institutional Review Board of the AOU Mater Domini/University Magna Graecia in the meeting of May 21st 2014.

### Tumor samples

Tumor samples and matched normal mucosa were obtained from patients referring to General Surgery Unit of University Hospital Magna Graecia of Catanzaro (Catanzaro, Italy), who had undergone surgical resection for colon cancer. Biopsies were immediately snap frozen and stored at -80°C. All diagnoses were confirmed by reviewing of hematoxylin/eosin-stained tissue sections.

### Patient’s demographics

General demographic information, histo-patological and clinical parameters, surgical treatment and follow-up data were collected prospectively. We summarize below the clinical characteristics of the patients included in the study. Among the 37 patients, 13 were women and 24 were males. Mean age of patients was 68 years old (range 47–84). Stage was known for 36 of the 37 patients: 6 patients had stage I disease, 13 patients had stage II disease, 12 patients had stage III disease and 5 patients had stage IV disease. Grade was known for 35 out of 37 patients: 26 patients had tumors that were graded G2 and 9 patients had tumors that were graded G3. Of the patients included in the present study, four presented distant metastasis. None of the patients received chemotherapy or radiation therapy prior to surgery. See Supplementary Table 1.

### DNA extraction and quality assessment

DNA was extracted from normal and pathological tissues and from blood using the PureLink® Genomic Kit (Invitrogen, Carlsbad, CA, USA) following the manufacturer’s instructions. DNA quality and quantity were assessed using the Qubit Fluorometer (Invitrogen, Carlsbad, CA, USA) and the 2200 Tape Station instrument (Agilent Technologies, Inc, Santa Clara, CA, USA). Good quality genomic DNA was subjected to library preparation prior to sequencing.

### Next generation sequencing

NGS was conducted using the Ion AmpliSeq™ Comprehensive Cancer Panel on the Ion Torrent platform (Thermofisher, MA, USA). The Ion AmpliSeq™ Comprehensive Cancer Panel provides complete exon coverage of 409 cancer-associated genes. We used the Ion AmpliSeq Library Kit 2.0 for library preparation, the Ion PI™ OT2 kit v2 and Ion OneTouch-2 Instrument for emulsion PCR and template preparation, and the Ion PI™ Sequencing 200 kit v3 with the Ion PI™ v2 Chip and Ion Proton Sequencer as the sequencing platform (Thermofisher). Libraries were generated from 40ng of input genomic DNA as measured by Qubit 2.0 Fluorometer (Thermofisher). Samples were considered eligible for sequencing if positive Ion sphere particles were >70% after template enrichment. For each Ion PI™ v2 Chip 4 samples were loaded. Samples were barcoded using Ion Xpress Barcode Adapters (Thermofisher).

### Bioinformatics analysis

Data obtained from NGS were processed using Torrent Suite software to generate sequence reads, trim adapter sequences, filter and remove poor signals and low quality reads. Variants detected within the 16,000 amplicons of the 409 genes of the panel were analyzed by the Torrent Variant Caller and the Torrent Suite Software v5.0.5 using the ‘Variant Caller v5.0.3.5’ plug-in (Thermofisher). The parameters used to filter the variants were coverage, quality and frequency. The parameters set for tumor samples were: coverage ≥ 50, quality score ≥ 30 and frequency ≥5%. The parameters set for PBL and normal mucosa were: coverage ≥ 10 and quality score ≥ 20. To remove common germ-line variants, tumor variants were filtered through the variants obtained from a pool of peripheral blood samples from some of the patients under analysis (n = 13). The candidate somatic variants were further filtered through the dbSNP141 and the 1000 Genomes Project datasets. The resulting variants were annotated according to the Catalogue of Somatic Mutations in Cancer (COSMIC) database as COSMIC variants. Finally, the potential damaging effects of the identified variants were evaluated through the prediction algorithms SIFT [63] and Polyphen2 [64].

### Statistical analysis

Analysis of the median number of mutations in tumors arising in different colon segments was performed by Student’s *t*-test. Significance of associations between mutated genes and clinical-pathological parameters was evaluated using Fisher’s exact test or Chi-square test.

Significance of associations between mutated genes and tumor localization was evaluated by a two-step statistical analysis that included a prior analysis of the associations by the Chi-square test followed by individual validation with Fisher’s test.

Survival curves were estimated using the Kaplan–Meier method and compared using the Log Rank test. OS was calculated from the day of surgery to the day of death or to the end of follow-up. Univariate survival analysis with calculation of Hazard Ratios (HR) was performed using Cox’s proportional-hazards model.

Multivariate survival analysis was performed using a Cox’s multiple linear regression model based on Firth’s bias correction method. A p-value ≤0.05 was considered to be statistically significant. R statistical environment was used to conduct the multiple statistical analyses [65].

### Sanger sequencing

Sanger sequencing was performed using BigDye terminator v3.1 (Thermofisher) with ABI 3100 Genetic Analyzer (Thermofisher).

## SUPPLEMENTARY MATERIALS FIGURES ANS TABLES










