# Supplementary material for: Identification of different mutational profiles in cancers arising in specific colon segments by next generation sequencing
Source: Oncotarget. 2018 May 8;9(35):23960–74. doi: 10.18632/oncotarget.25251 (PMC5963617; doi:10.18632/oncotarget.25251)
Supplement: Supplementary file 1 [file oncotarget-09-23960-s001.pdf]

Identification of different mutational profiles in cancers arising in specific colon segments by next generation sequencing

SUPPLEMENTARY MATERIALS

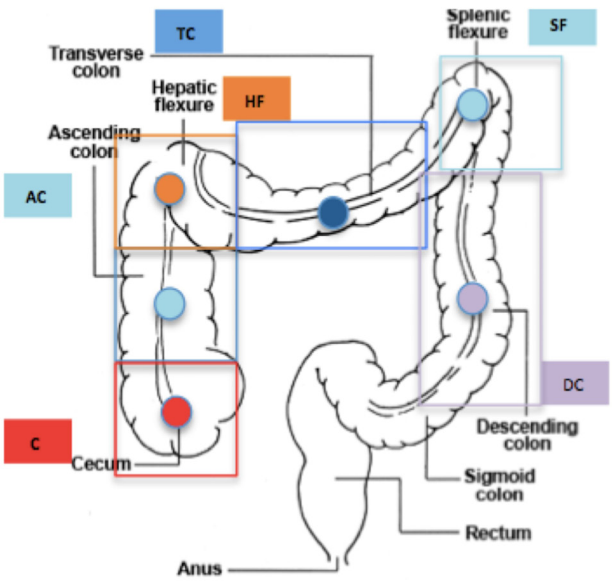

Supplementary Figure 1: Schematic representation of the different colon segments in which tumors under analysis were resected. **AC**, ascending colon, **BC**, descending colon, **HF**, hepatic flexure, **SF**, splenic flexure, **TC**, transverse colon and **C**, cecum.

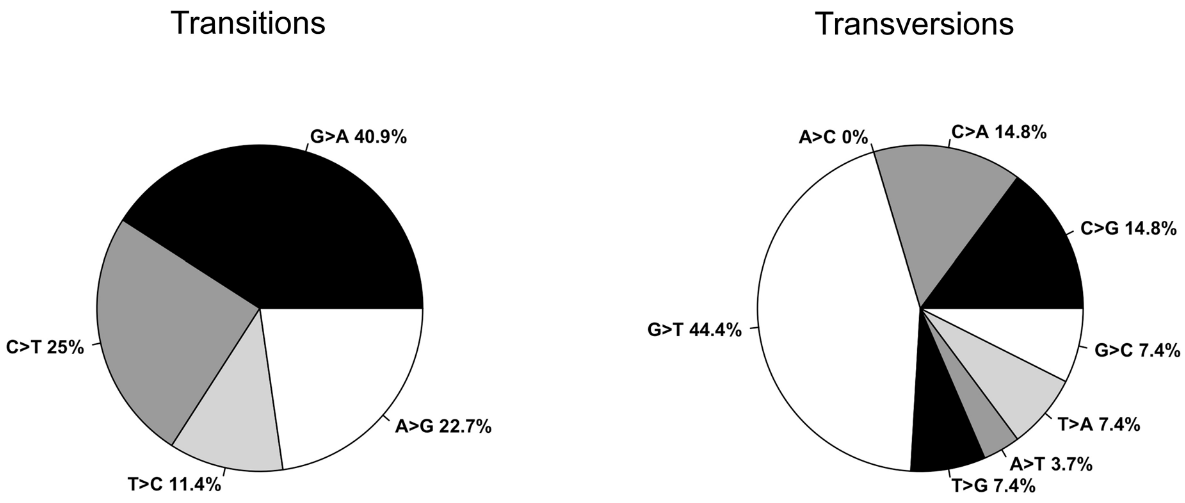

Supplementary Figure 2: Classification of variants identified in cancers arising in the cecum.

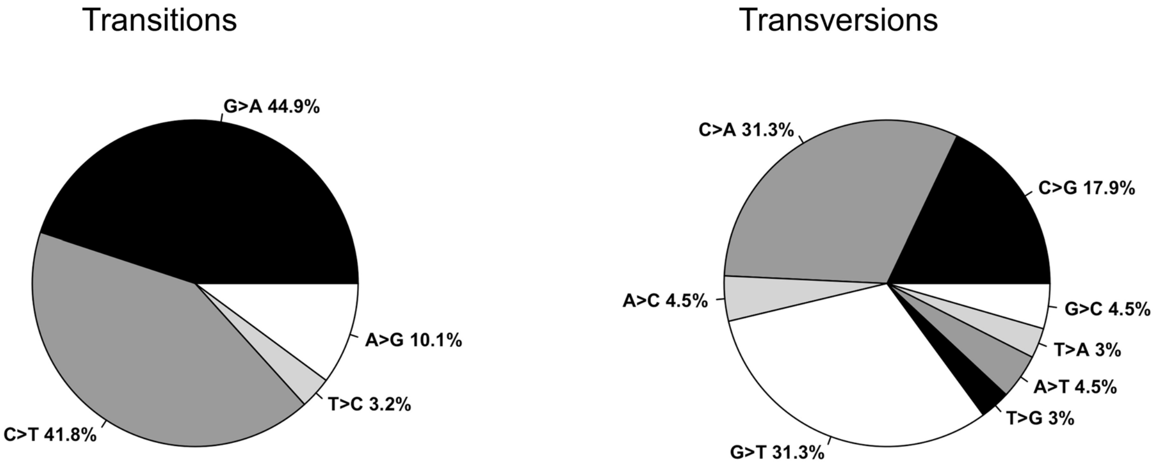

Supplementary Figure 3: Classification of variants identified in cancers arising in ascending colon.

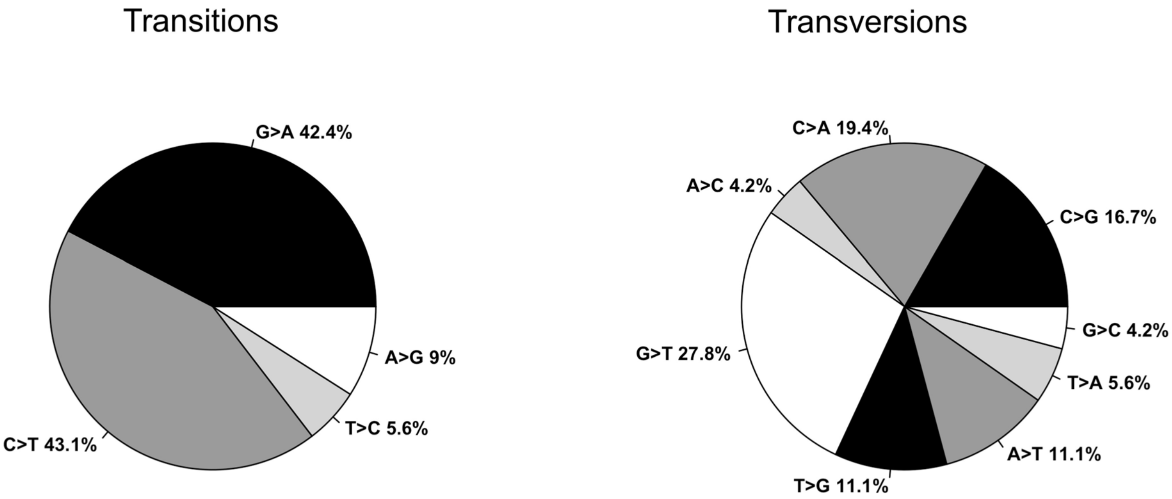

Supplementary Figure 4: Classification of variants identified in cancers arising in hepatic flexure.

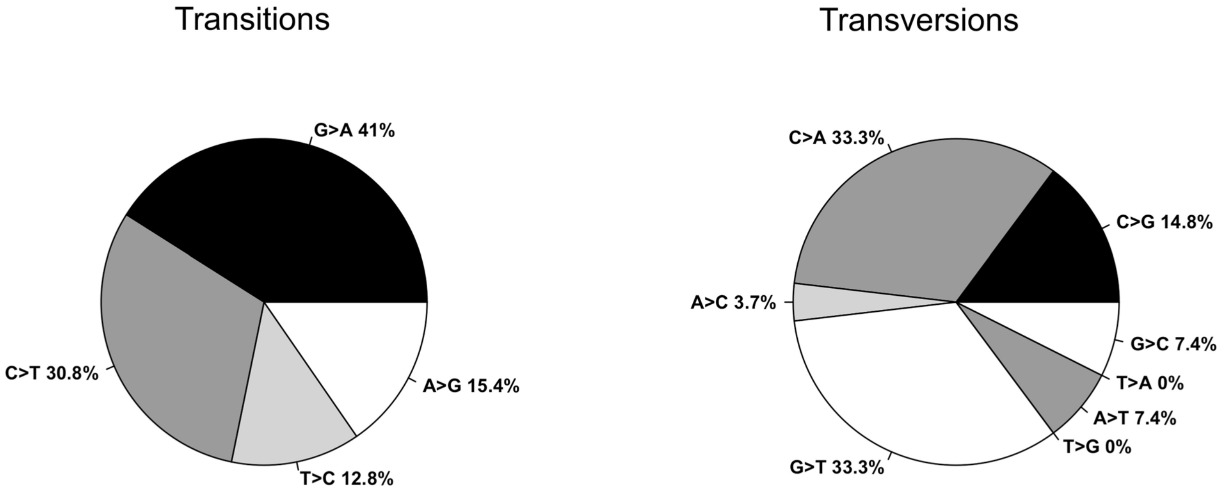

Supplementary Figure 5: Classification of variants identified in cancers arising in transverse colon.

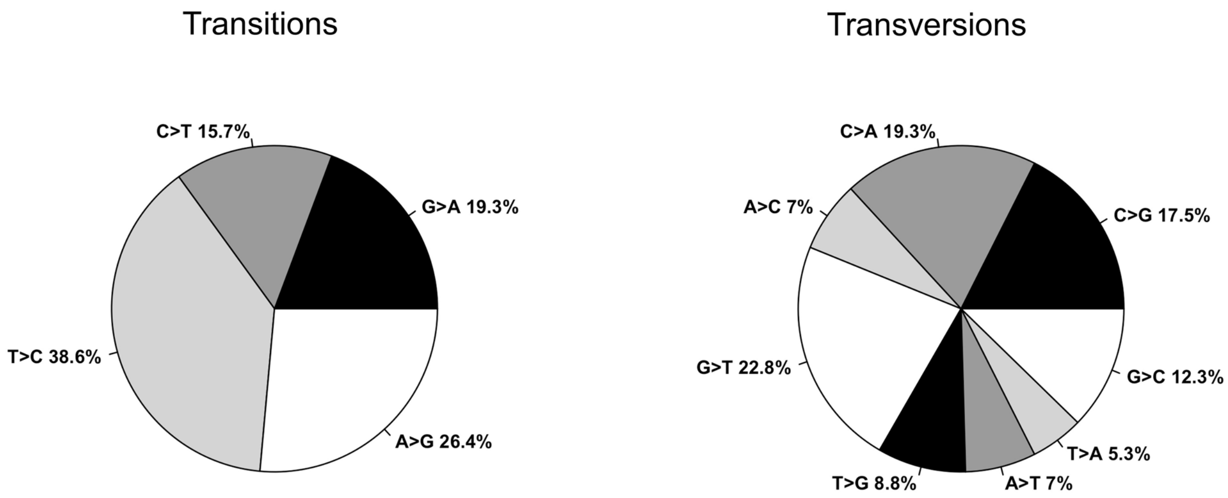

Supplementary Figure 6: Classification of variants identified in cancers arising in splenic flexure.

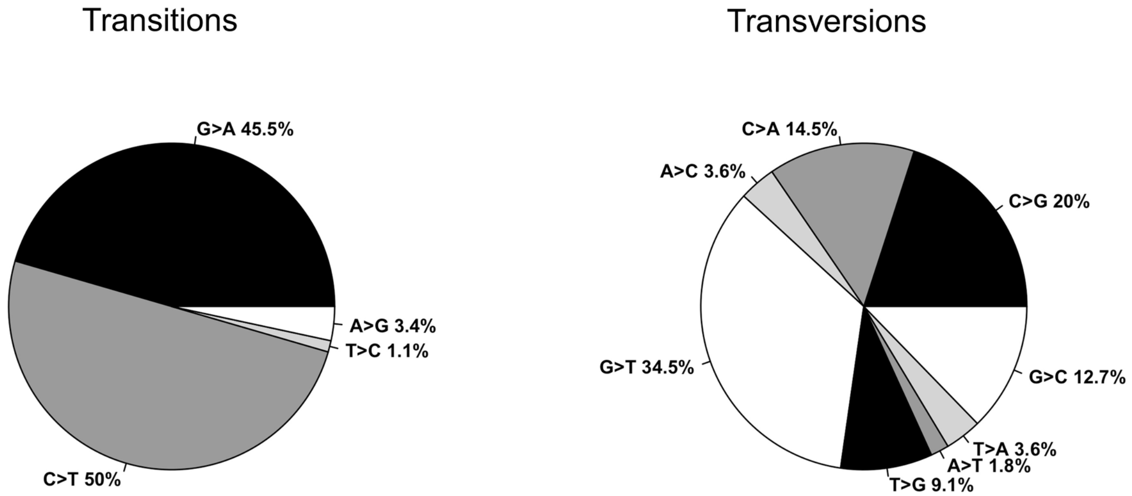

Supplementary Figure 7: Classification of variants identified in cancers arising in descending colon.

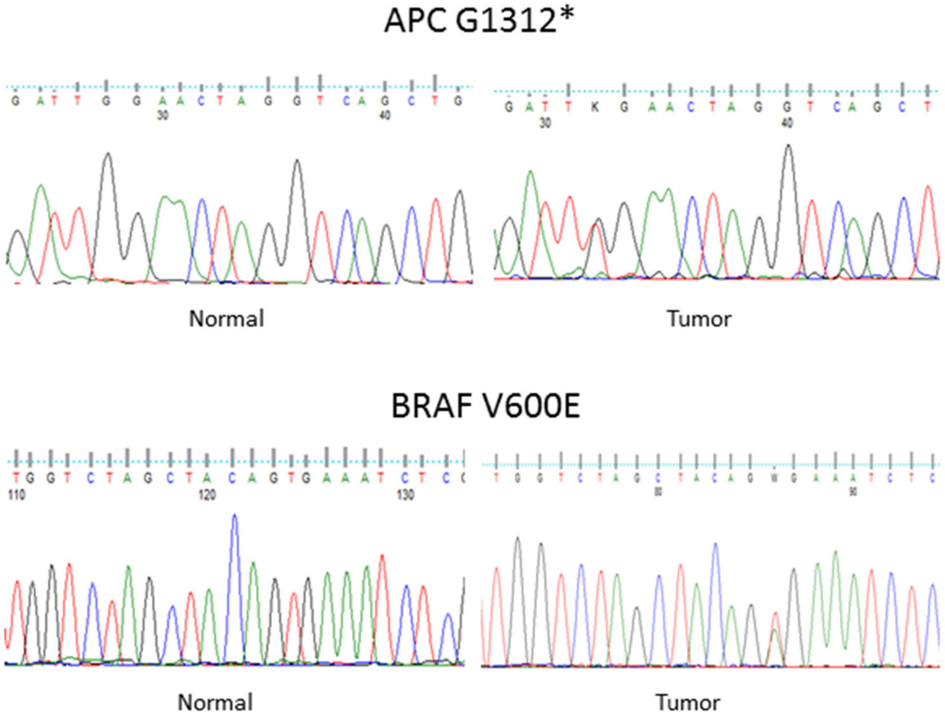

Supplementary Figure 8: DNA Sanger sequencing on matched normal and cancer samples of CC12 and CC28 patients in which are shown the V600E variant of BRAF and the G1320* variant of APC. (A) Left, wild type APC sequence from normal tissue of patient CC28; right, APC G1312* mutation in patient CC28. **(B)** Left, wild type BRAF sequence from normal tissue of patient CC12; right, BRAF V600E mutation in patient CC12.

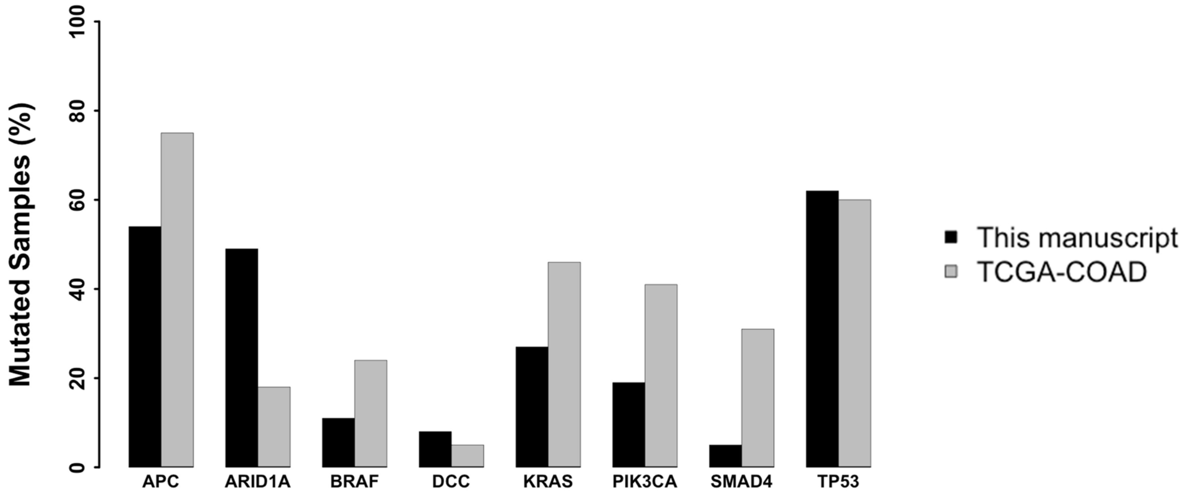

Supplementary Figure 9: Comparative analysis of the mutation frequency in 8 genes commonly associated with CRC.

Mutations identified in genes commonly mutated in colon cancer

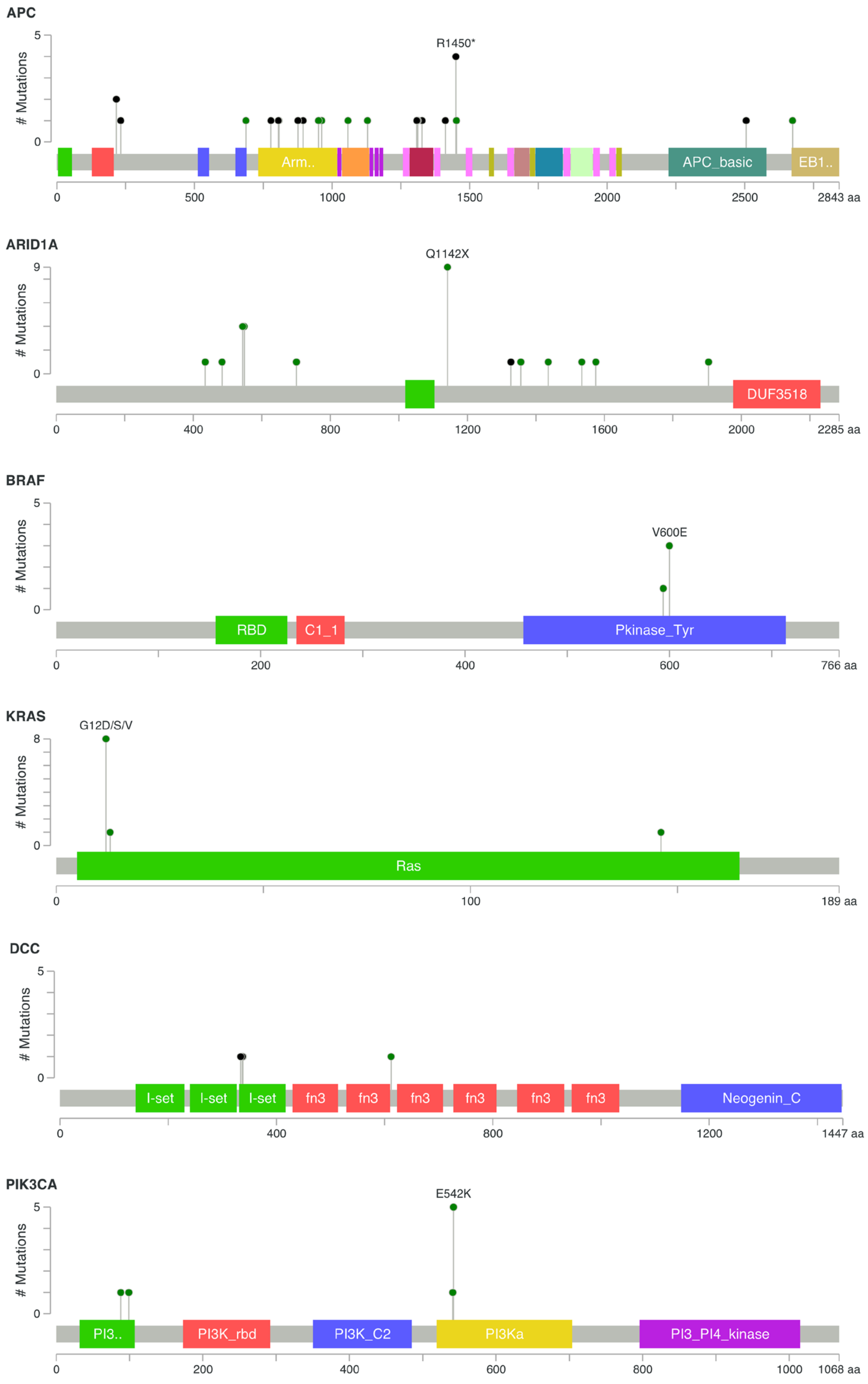

Supplementary Figure 10: Mutation mapper plots showing the position of amino acid changes corresponding to the variants identified within 8 commonly mutated genes in colorectal cancer.

Mutations identified in genes commonly mutated in colon cancer

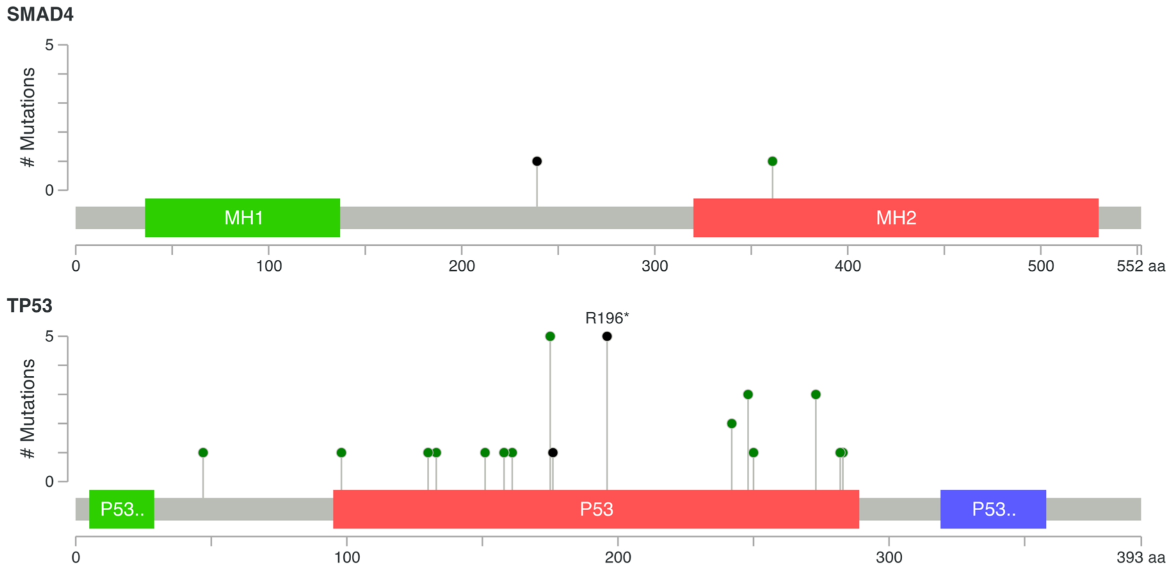

Supplementary Figure 11: Mutation mapper plots showing the position of amino acid changes corresponding to the variants identified within 8 commonly mutated genes in colorectal cancer.

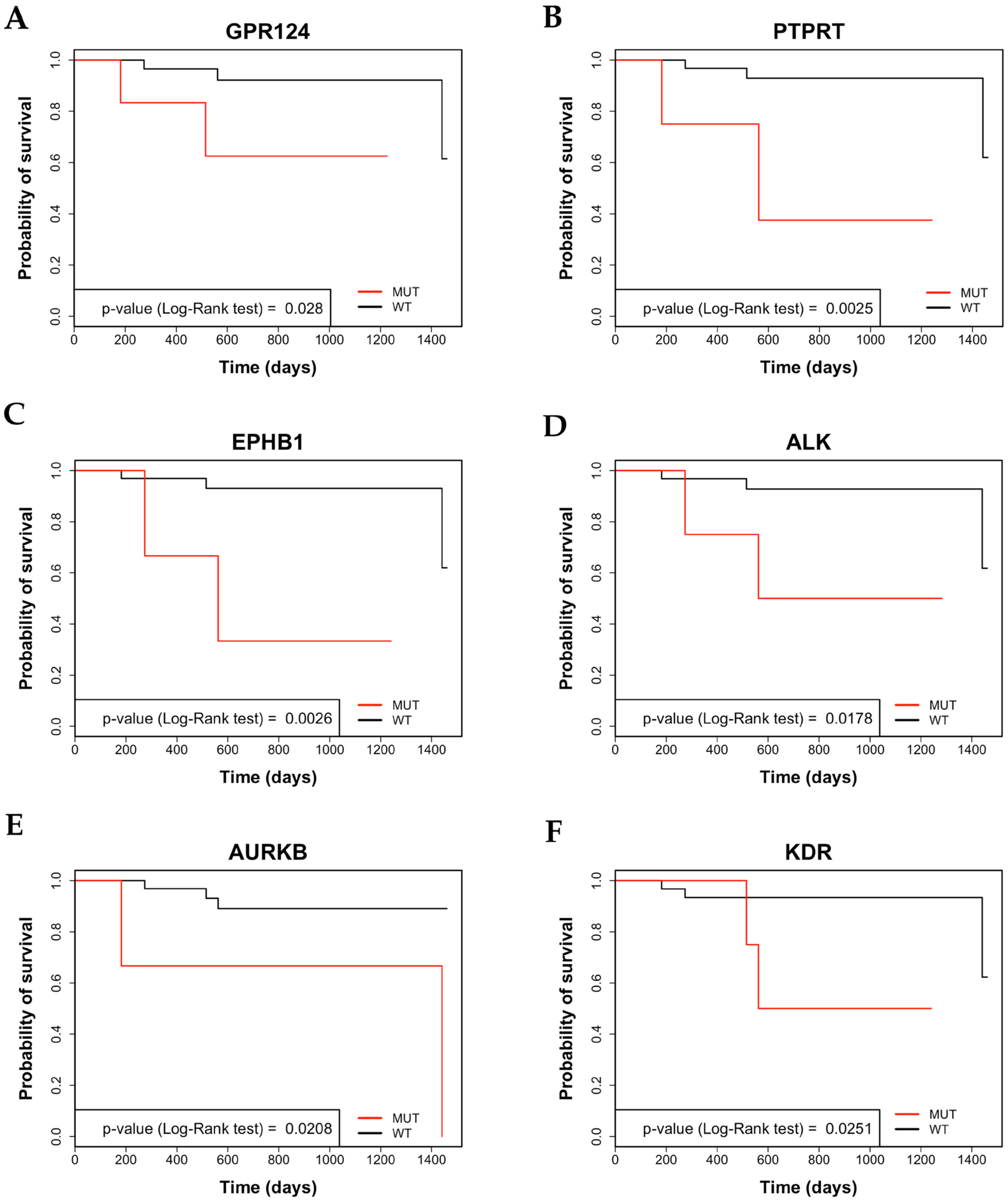

Supplementary Figure 12: Analysis by Kaplan-Meier curve of 4-year survival in colon cancer patients. **(A)** Kaplan-Meier survival curve of OS in CC patients that were stratified for the presence of variants in GPR124. **(B)** Kaplan-Meier survival curve of OS in CC patients that were stratified for the presence of variants in PTPRT. **(C)** Kaplan-Meier survival curve of OS in CC patients that were stratified for the presence of variants in EPHB1. **(D)** Kaplan-Meier survival curve of OS in CC patients that were stratified for the presence of variants in ALK. **(E)** Kaplan-Meier survival curve of OS in CC patients that were stratified for the presence of variants in AURKB. **(F)** Kaplan-Meier survival curve of OS in CC patients that were stratified for the presence of variants in KDR.

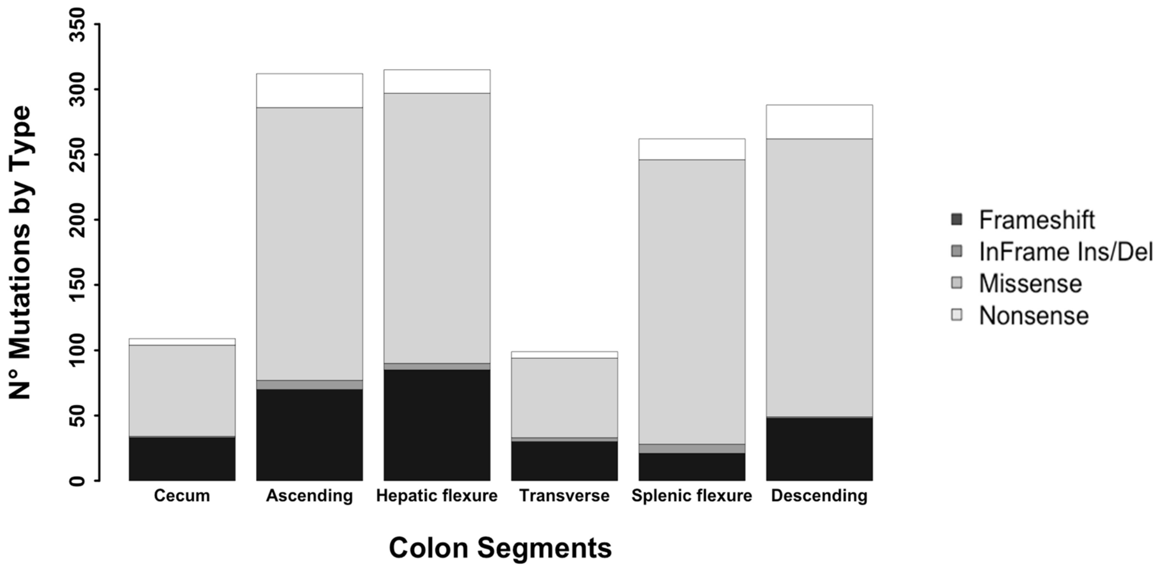

Supplementary Figure 13: Distribution of variants in tumors stratified by anatomical site. Bar plot showing the distribution of missense, nonsense, small indels and frameshift variants in tumors arising in different anatomical sites. Tumors originating in the splenic flexures presented more variants/tumor (median value=52, range 16-112) compared with tumors originating in other sites and in particular with those originating in the cecum (median value=18, range 6-35) or in the transverse colon (median value=25, range 7-37), respectively. In addition, the comparison of mutation profiles exhibited by tumors originating in different colon segments also highlighted significant qualitative differences in the type of variants identified. Tumors originating in the splenic flexure presented the highest average number of missense SNVs (median value=41) compared with those originating in the cecum (median value=11.5) or in the transverse segment of the colon (median value=17.5). On the other hand, tumors that originated in the cecum and in the splenic flexure presented the lowest number of frameshift-type variants (median values=4.5 and 5, respectively) in comparison with tumors originating in other colon sites (median values=9 in ascending colon and 10.5 in hepatic flexure, respectively).

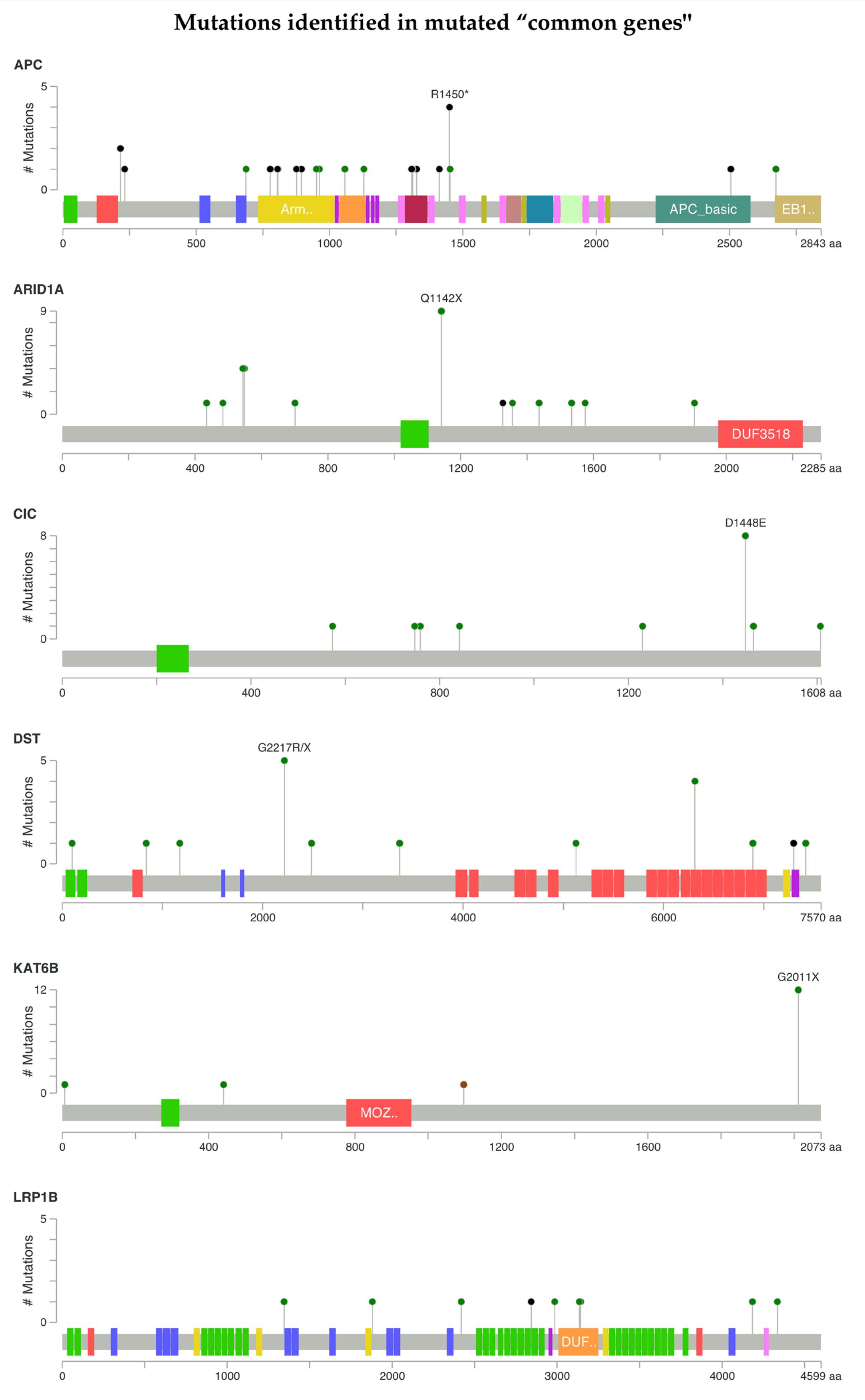

Supplementary Figure 14: Mutation mapper plots showing the position of amino acid changes corresponding to the variants identified within the "common" genes.

Mutations identified in mutated "common genes"

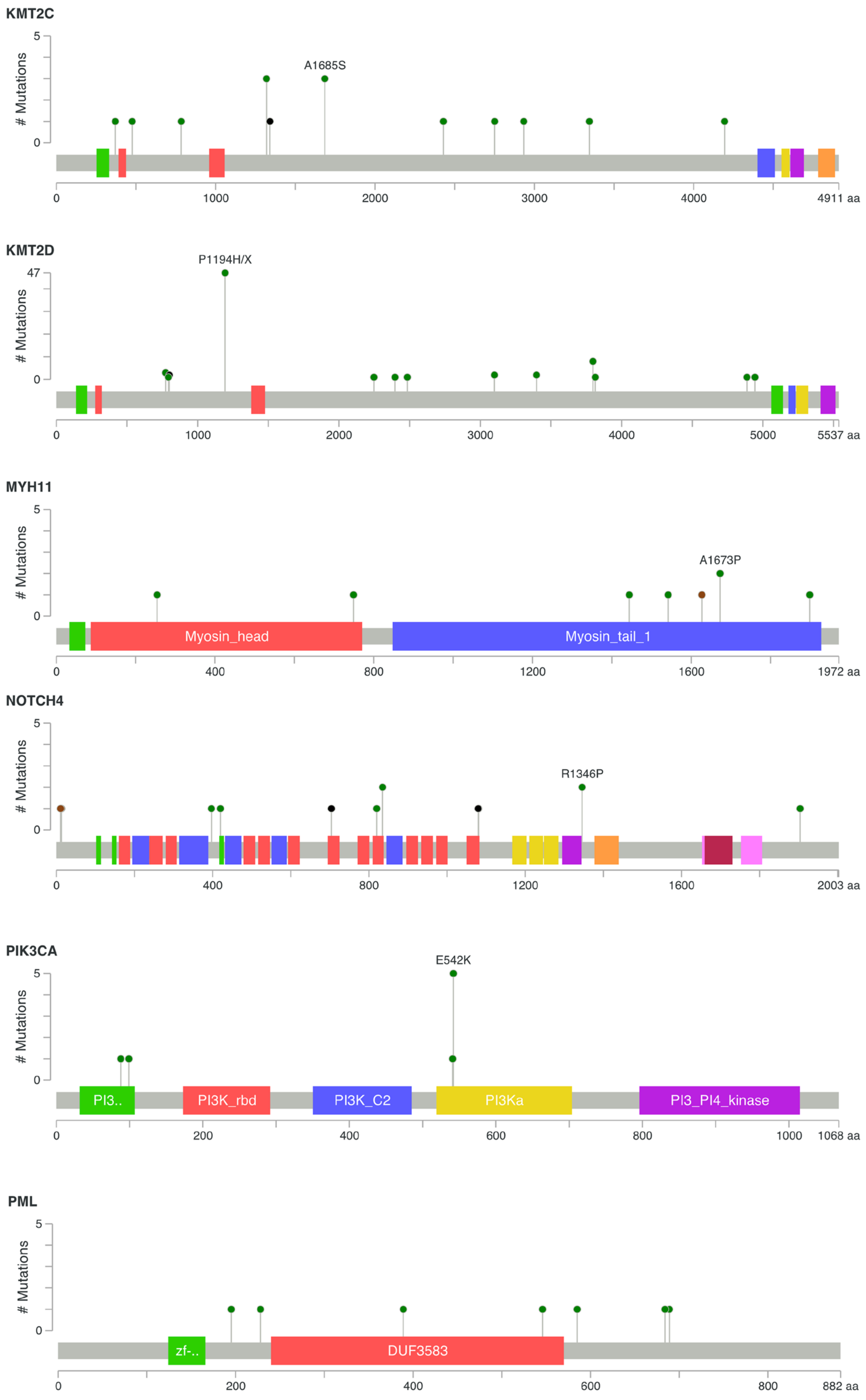

Supplementary Figure 15: Mutation mapper plots showing the position of amino acid changes corresponding to the variants identified within the "common" genes.

Mutations identified in mutated “common genes"

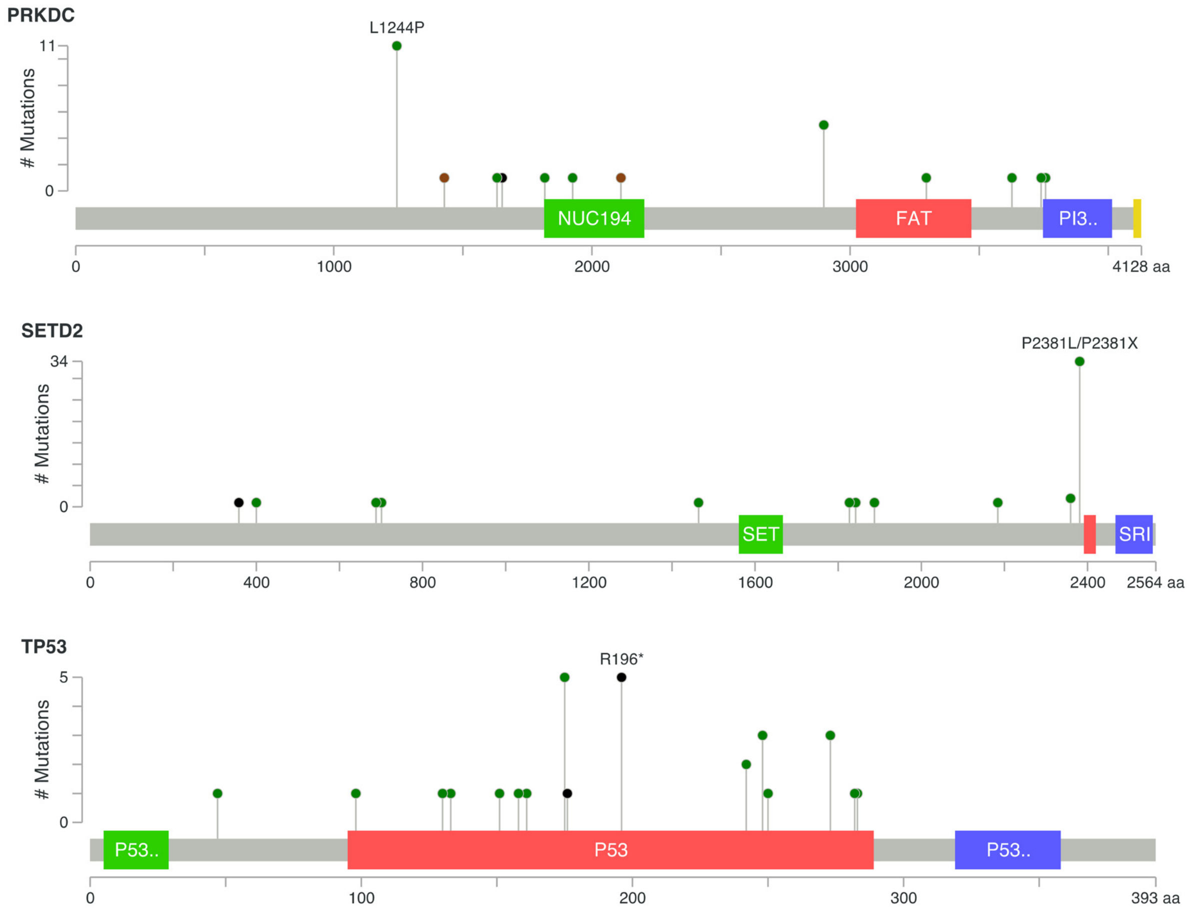

Supplementary Figure 16: Mutation mapper plots showing the position of amino acid changes corresponding to the variants identified within the “common” genes.

Mutations identified in «site-associated genes» in colon cancer

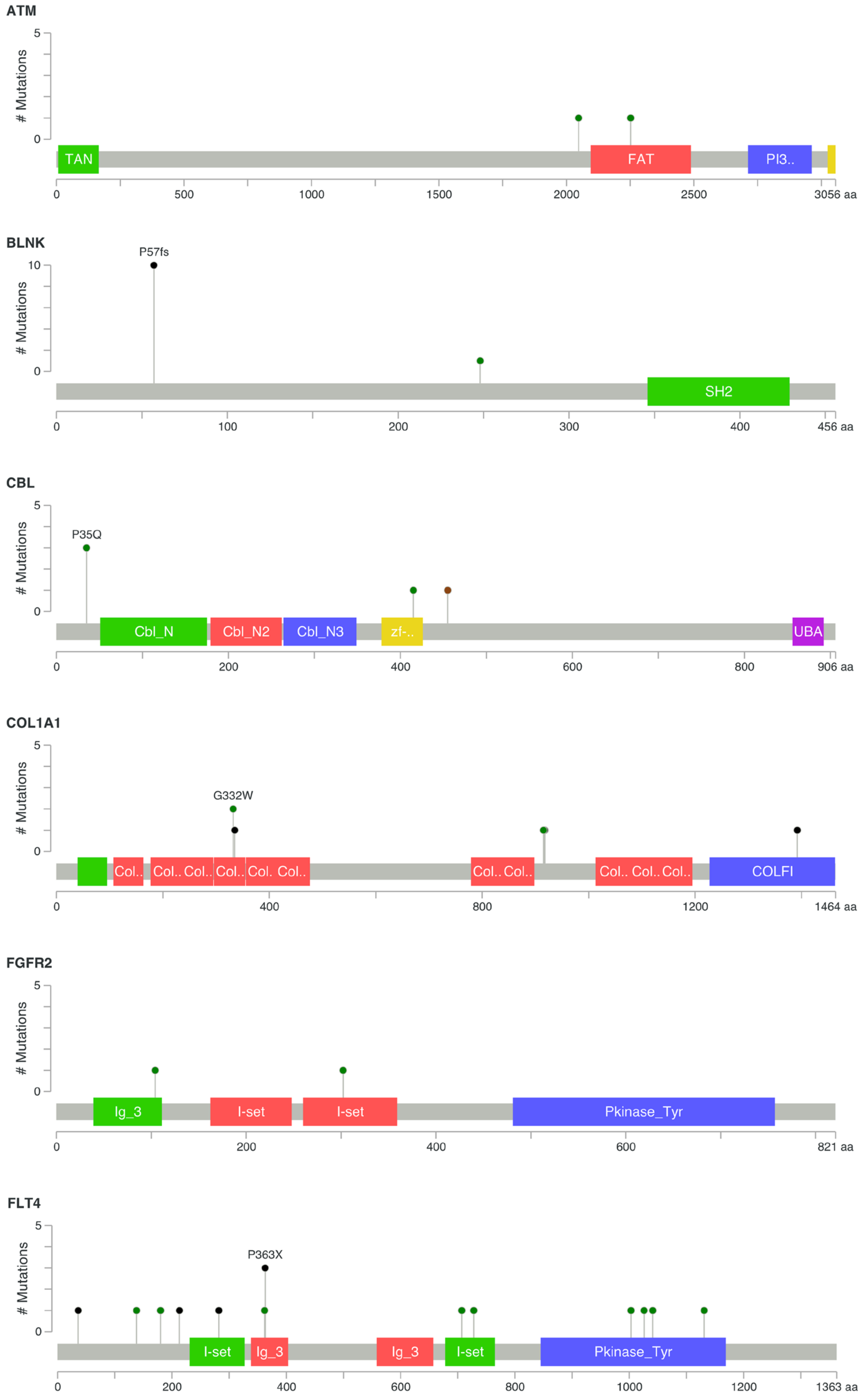

Supplementary Figure 17: Mutation mapper plots showing the position of amino acid changes corresponding to the variants identified within the "site-associated" genes.

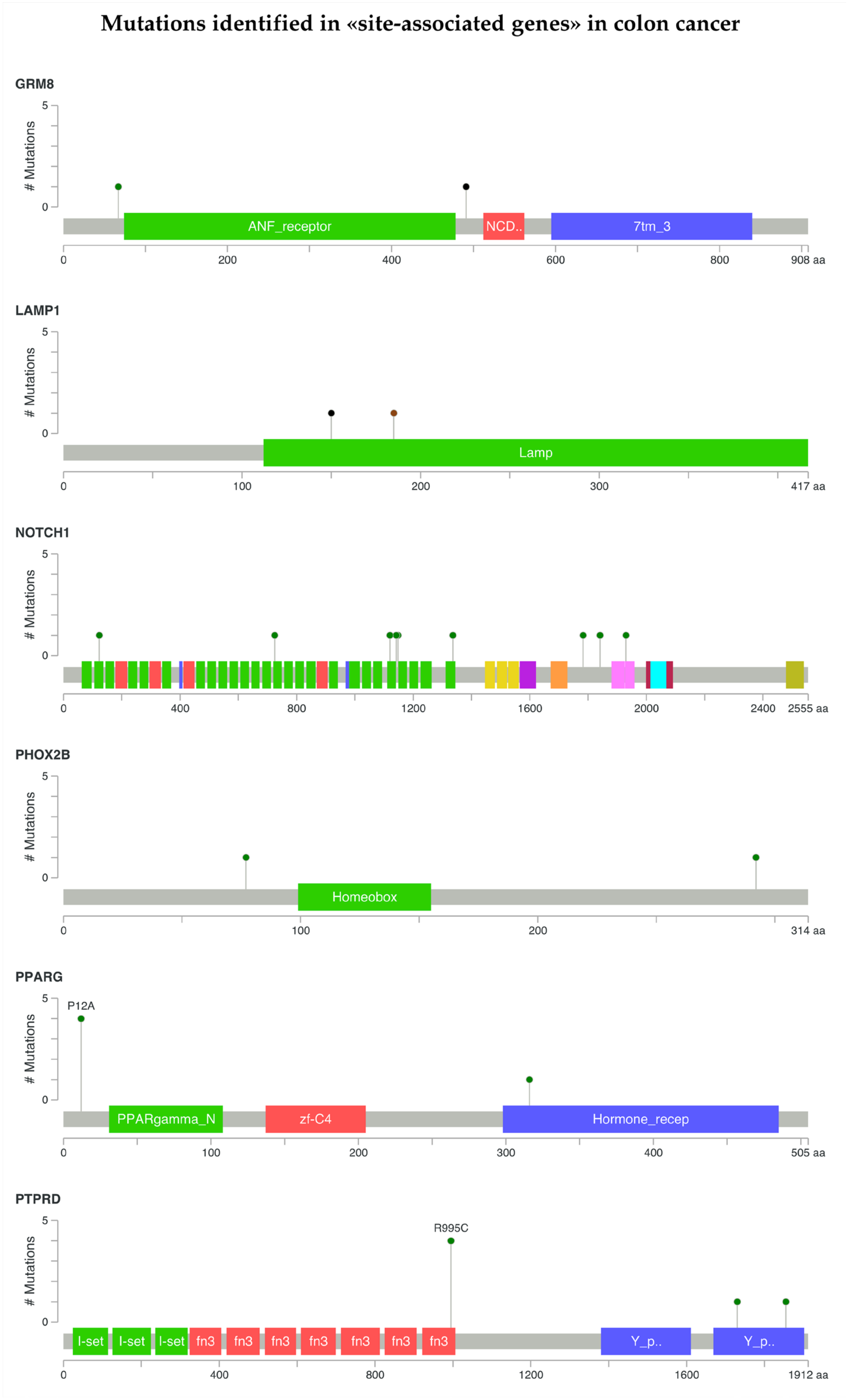

Supplementary Figure 18: Mutation mapper plots showing the position of amino acid changes corresponding to the variants identified within the "site-associated" genes.

Mutations identified in «site-associated genes» in colon cancer

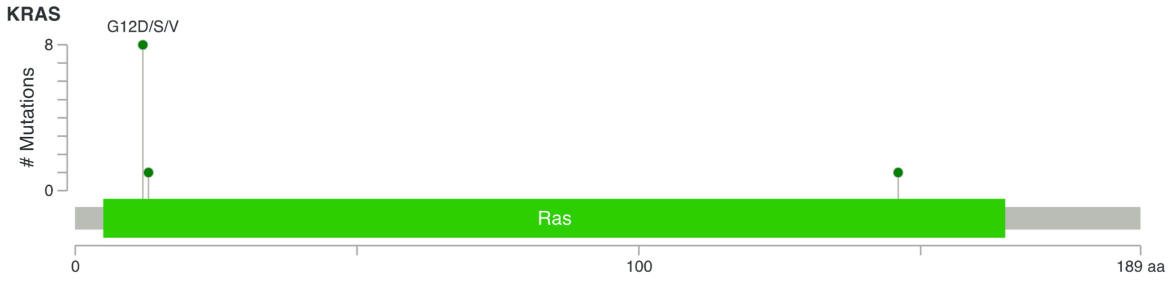

Supplementary Figure 19: Mutation mapper plots showing the position of amino acid changes corresponding to the variants identified within the "site-associated" genes.

Supplementary Table 1: Clinical-pathological characteristics of the 37 colon cancer patients included in the study.

See Supplementary File 1

Supplementary Table 2: Comprehensive list of the variants identified in the 37 colon cancer samples analyzed.

See Supplementary File 2

Supplementary Table 3: List of COSMIC variants identified in the 37 colon cancer samples analyzed.

See Supplementary File 3

Supplementary Table 4: Association between mutated gene and clinical variables were assessed using contingency tables with Chi-square test.

See Supplementary File 4
